# Neural Population-Level Memory Traces in the Mouse Hippocampus

**DOI:** 10.1371/journal.pone.0008256

**Published:** 2009-12-16

**Authors:** Guifen Chen, L. Phillip Wang, Joe Z. Tsien

**Affiliations:** Brain and Behavior Discovery Institute and Department of Neurology, School of Medicine, Medical College of Georgia, Augusta, Georgia, United States of America; University of New South Wales, Australia

## Abstract

One of the fundamental goals in neurosciences is to elucidate the formation and retrieval of brain's associative memory traces in real-time. Here, we describe real-time neural ensemble transient dynamics in the mouse hippocampal CA1 region and demonstrate their relationships with behavioral performances during both learning and recall. We employed the classic trace fear conditioning paradigm involving a neutral tone followed by a mild foot-shock 20 seconds later. Our large-scale recording and decoding methods revealed that conditioned tone responses and tone-shock association patterns were not present in CA1 during the first pairing, but emerged quickly after multiple pairings. These encoding patterns showed increased immediate-replay, correlating tightly with increased immediate-freezing during learning. Moreover, during contextual recall, these patterns reappeared in tandem six-to-fourteen times per minute, again correlating tightly with behavioral recall. Upon traced tone recall, while various fear memories were retrieved, the shock traces exhibited a unique recall-peak around the 20-second trace interval, further signifying the memory of time for the expected shock. Therefore, our study has revealed various real-time associative memory traces during learning and recall in CA1, and demonstrates that real-time memory traces can be decoded on a moment-to-moment basis over any single trial.

## Introduction

What are neural population-level memories in real-time? Can we decode neural populations' memory traces as the subjects form and recall memories? How meaningfully can real-time memory traces in the brain be related to a behavioral readout? These enduring questions have attracted a keen interest of many philosophers, psychologists, neuroscientists and beyond [1]–[3]. Great progresses have been toward our understanding of memory processes at the molecular, synaptic, anatomical, and single neuron levels in the past decades [4]–[8]. Yet, real-time transient dynamics of neuronal population-level memory traces still remains unknown.

The CA1 region of the hippocampus is well known to be a crucial site for processing associative memories which typically contain information about what, where, and when major events occurred. One of the classical associative memory tasks widely used by researchers is the trace conditioning memory paradigm [3], [7]–[12]. There are several variations in term of the nature of conditioned (tone, light, odor, etc) and unconditioned stimuli (mild electrical foot-shock, air-puff to eyelid, etc). These protocols can produce long-lasting associative memories in the brains of various animal species. In a typical trace conditioning paradigm, the presentations of a preceding conditioned stimulus (CS), such as a neutral tone, and a subsequent unconditioned stimulus (US), such as mild foot-shock to foot or air-puff to eyes, are separated in time by an inter-stimuli interval [8]–[12]. With repetitions of paired CS-US presentations in a given environment, humans and laboratory animals can form the rich associative memories of events and their temporal relationship, as well as the context in which the associative events take place. It has been shown that this classical Pavlov conditioning induces increased neuronal discharges in the hippocampus and cerebellum [3], [7], [9]–[11]. Such learning paradigms have also been shown to produce changes in latency responses and membrane potentials [3], [9]–[15]. The well-defined, salient nature of cues and memory produced by this classic conditioning test offers an excellent paradigm for search of the neural basis of memory traces [3].

As a prevalent phenomenon in the brain, large response-variability in single neuron activity within any single trial typically makes it difficult to reliably decode the neural activity patterns on a moment-to-moment temporal basis in a given single trial. That is, the trial-to-trial variability of single neuron responses (activity changes of a single neuron) does not allow the reliable prediction of, and consequently, understand whether and/or how brains form snap-shot memory representation of events at any given moment in time. In another words, spike raster plots, while being quite useful to determine neurons' response-selectivity, are not a very effective approach to decoding real-time memory patterns. Moreover, data-averaging practice over trials intrinsically limits the analysis to a selected single time point (say, marked by the presentation of the conditioned stimulus during the recall tests as time zero). This would inevitably lead to loss of most crucial information about memory traces if they are reappeared in tandem with variable intervals across different trials. Therefore, one of the major goals for the memory field is to understand how memory is represented as transient dynamic patterns at the neuronal population level on a moment-to-moment basis and then to demonstrate the explicit relationship between those neural patterns with a behavioral readout during learning and recall.

Since brains are likely to achieve real-time representation of memories through the coordinated activity of ensemble neurons [16], [17], we employed our newly developed large-scale ensemble recording techniques to simultaneously measure the activity patterns of large numbers of neurons in the CA1 region of the mouse hippocampus when animals were learning and retrieving various associative memories. We used the trace-fear conditioning protocol to produce trace-fear memory as well as contextual fear memory. A wide range of lesion studies on mice, rats, monkeys, and humans all suggest that both contextual and trace fear conditioning memory requires the structural integrity of the hippocampus [2], [7]–[15], [18]–[24]. This classic Pavlov learning paradigm allows us to directly examine the ensemble patterns in the hippocampus in responses to explicit cues as the CA1 population engages in multiple stages of memory processing. More importantly, this classic memory test can further enable us to assess the causal relationship between these memory traces and behavioral performances during the acquisition and retrieval of associative memories.

## Results

### Encoding Patterns Produced by Trace Conditioning Stimuli

**Figure 1:**
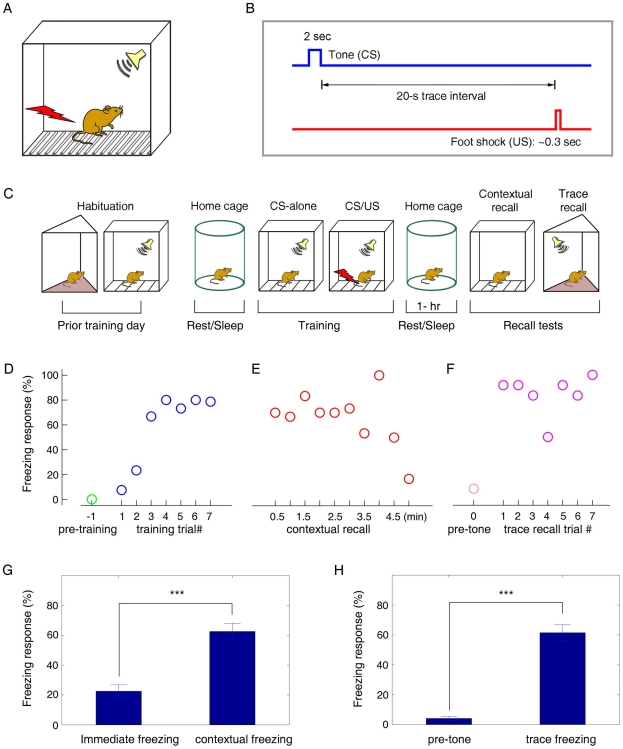
Associative trace fear conditioning. (**A**) Illustration of a fear conditioning chamber for producing a tone-shock trace fear memory. A neutral tone precedes a mild foot-shock via a metal grid floor. (**B**) The trace interval between the offset of the tone (2 sec) and the onset of the foot shock (0.3 sec) is 20 seconds. (**C**) Schematic representation of experimental paradigm for trace fear conditioning. On the training day, the experiment begins as follows: one-hour pre-training sleep/rest in home cage; pre-training exploration in the conditioning chamber; trace conditioning in the conditioning chamber; one-hour post-training rest/sleep in home cage; contextual recall in the conditioning chamber; trace conditioning recall in a novel chamber. (**D**) Immediate freezing in mouse #1 increased as the CS-US pairing repeated during the training phase. (**E**) Contextual freezing in the 1-h contextual retention test suggests the contextual fear formation in mouse #1; at the end of the 5-min test, freezing decreased as there was no reinforcement shock. (**F**) Robust trace freezing upon the recall tone. The tone was presented for seven times (trials) with a 1-min interval. (**G**) As a group (n = 10), there was a significant contextual freezing as revealed by the 1-h contextual retention test (62.5%±5.5%, p<0.001). (**H**) The same set of mice showed a significant increased freezing during the 1-h trace retention test (n = 10, 61.5%±5.3%, p<0.001).

We used the trace fear conditioning paradigm to train ten adult, wild-type male mice (5–6 month old). Our trace conditioning contained a neutral tone (CS, 85dB, 2 sec) which was followed by a delayed mild foot shock (US, 0.75 mA, 0.3 sec) delivered via a metal grid floor in a fear conditioning chamber (Fig. 1A). The fixed time interval, or “trace” interval, between the CS and the US was 20 seconds (Fig. 1B). This long-duration trace conditioning is extremely sensitive to hippocampal-lesion animals [3], [8], [18]–[23]; it can produce various memories such as the memories for the shock event, the causal relationship and timing information of the tone and shock, as well as the corresponding environment. Our experiments started with a three-day habituation session prior to training, in which the mice were introduced to two distinct chambers (5 minutes in each chamber, per day). One chamber was for fear conditioning (learning) and the other for a one-hour trace fear memory retention test (recall). It has been shown that this type of pre-exposure to the context can ensure the formation and recall of specific contextual memory [25], [26]. We then show that seven repetitions of CS-US parings produced an increasing amount of immediate freezing during memory acquisition (Fig. 1D). At the one-hour contextual retention test, when the mice were brought back to the original conditioning chamber, the animals exhibited significant freezing during the 5-min contextual recall (Fig. 1E), indicating robust contextual fear memory. However, freezing tapered off by the end of the retention test, reflecting adaptive behavioral changes in the absence of US reinforcement.

After the contextual retention test, the mice were returned to the home cage for a brief break and then were placed into a novel chamber (recall chamber) for the trace retention test. As expected, the animals showed active exploratory behaviors in the recall chamber during the 3-min pre-tone period (Fig. 1F). However, as soon as the conditioned tone (2-sec) was played, the animals would freeze immediately and often remained in the freezing state for a significant period. During trace recall, the 2-sec tone was presented to the mice seven times with a 1-min interval, and the animals showed robust tone-induced freezing (Fig. 1F). The group data further confirmed that our long-duration trace conditioning protocol was highly effective in eliciting the formation of robust contextual fear memories (Fig. 1G, p<0.001) as well as trace fear-conditioning memories in mice (Fig. 1H, p<0.001). On the contrary, the unpaired CS/US protocol was ineffective in producing trace conditioning memory in our animals (Fig. S1), which is highly consistent with the well documented observations in literature [e.g. see 3], [7], [8], [11].

Using the large-scale *in vivo* recording technique that we have recently described for monitoring neural ensemble activity in the brain of freely behaving mouse [27]–[29], we set out to simultaneously record the activity of over two hundred neurons from the CA1 region of the mouse hippocampus as the animal underwent the acquisition and retrieval of fear memory. We first confirmed that our recordings were taken place in the CA1 region of the hippocampus by both post-experiment histological staining of the electrode positions (Fig. S2A) and unique CA1 physiological markers (e.g. ripples) (Fig. S2B and 2C). Furthermore, we confirmed our recordings were stable throughout the entire recording experiments based on the comparisons of the waveforms for the spikes recorded at the beginning and end of the experiments (Fig. S3 for putative excitatory units; and Fig. S4 for putative interneurons). The consistent waveforms during shock events also suggested that no electrical artifacts were taken for spikes (Fig. S3, S4).

**Figure 2:**
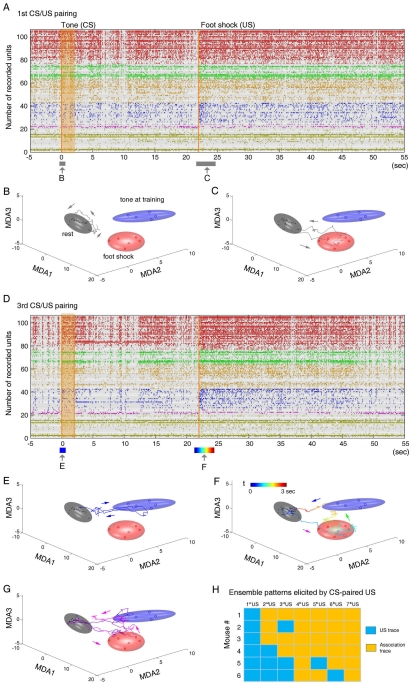
Ensemble dynamic patterns during learning. (**A**) Sixty-sec spike rasters of a selective set of CA1 units during the first CS-US pairing (105 units shown out of 208 simultaneously recorded units from mouse #1 for demonstration). The different colors represent several groups of cells with different response properties. The units listed at the bottom were selected from the non-responsive group (see details in SF 4). (**B**) MDA analysis shows ensemble patterns and dynamic trajectories in response to the first CS before the US arrived. The grey, blue and red ellipsoids represent the resting state, tone, and foot-shock clusters, respectively. The grey trajectory shows a transient (∼1-sec) ensemble dynamics at the moment marked by the arrow B near the zero time point in (A) in response to the first tone before the paired US arrived. This trajectory remained largely within the rest cluster, indicating that CA1 did not respond to the neutral tone initially. (**C**) A robust dynamic trajectory moving from the Rest cluster to Shock cluster was detected when the first paired US was delivered at the time point marked by the arrow C in (A). This transient trace lasted about 1.5 seconds. (**D**) The sixty-sec spike rasters demonstrate neural activity of the same set of cells shown in (A) during the third CS/US pairing. (**E**) The CS presentation in this third trial elicited a robust pattern (blue trajectory). The duration corresponds to the blue bar length in (D) marked by the arrow E. (**F**) The third paired US triggered an associative trajectory that transversed the shock cluster and the conditioned tone cluster. The color-coded trajectory, spanning for about 3 seconds, corresponds to the moment indicated by the colored bar in (D). (**G**) Two more examples of the unique US-CS associative trajectories elicited by foot shock at the fourth and sixth trials in the same animal (mouse #1) are shown here. The arrows indicate the moving directions of the ensemble trajectories. (**H**) The six-by-seven color matrix shows the occurrences of US-CS association patterns over seven trials from the six recorded mice. The blue squares indicate that foot shock (US) only triggered US ensemble trajectories early on, whereas the yellow squares indicate the US-CS association trajectory was elicited by foot shock, which became more prevalent after pairings.

An example of spike rasters for a subset of simultaneously recorded responsive units is shown (Fig. 2A, also see Fig. S5A). While the conventional spike raster histograms can provide some basic assessment of the response properties for recorded units by either averaging the responses of multiple units at a single trial (Fig. S5B) or by averaging the single-unit responses over multiple trials (Fig. S6A and C), it is difficult to use these simple-order methods or the visual inspection of spike rasters for the deeper and quantitative understanding of ensemble patterns and real-time dynamics of these simultaneously recorded units.

Thus, we employed a MDA method, a supervised dimensionality reduction-based statistical pattern classification method, to uncover the neuronal population level-encoding patterns from large-scale datasets [17], [28], [30]. Firing patterns during prior training rest period (gray ellipsoid), conditioned tone presentation, and foot shock epochs are shown after being projected to a three-dimensional space obtained by using MDA (Figure 2B). Each dot within an ellipsoid is a statistical result for the ensemble of the simultaneously recorded neurons from a single trial. The MDA representation of these ensemble responses was built from five trials for the CS ellipsoid (excluding the tone during the first pairing), for the US ellipsoid from 6 trials of US, and the rest ellipsoid from sampling 14 resting epochs (for matching 7 CS and 7 US epochs). The boundaries of each ellipsoid reflect the 2 sigma boundaries as we fitted the dot clusters with Gaussian distributions in the MDA space. We validated the MDA classification power by performing “test” using the remaining trials (1 to 3 trials left out of the training model). The “leave-one-out” validation method revealed that our statistical pattern classification can achieve up to 99% prediction accuracy (see Table S1). Thus, the MDA-based statistical pattern classification method enabled the qualitative and quantitative measurement of various population encoding patterns associated with conditioned stimuli and conditioned stimuli as distinct Gaussian ellipsoids in the MDA subspaces. With all experimental categories, those distinct patterns formed by these stimuli were well separated (see the all-category MDA plots in Fig. S7A and S7B).

In order to monitor the transient dynamics of CA1 ensemble activity patterns, we combined the MDA method with a sliding-window technique for assessing the temporal dynamics of neural processing. By using the fixed matrix coefficients produced by the MDA method, we computed the transient projection of neural ensemble responses throughout the recorded datasets with a 10-msec sliding window [17], [28], [30]. As such, the temporal evolution of the ensemble activity patterns could be directly visualized as transient dynamical trajectories in these encoding subspaces on the 10-msec to 10-msec time frame [28], [30]. The continuous plotting of these 10-msec dots would give rise to high temporal resolution dynamical trajectories in the MDA spaces. For example, upon the first tone (before the first paired foot shock arrived), the CA1 trajectory did not show any significant protrusion outside the Rest ellipsoid (Fig. 2B), indicating the neutral tone at this stage did not evoke significant ensemble responses in the hippocampus. On the contrary, the first foot-shock (20 seconds after the CS) elicited a robust CA1 ensemble trajectory that began in the Rest ellipsoid, and quickly visited the corresponding Shock ellipsoid (US cluster) before returning to the Rest (Fig. 2C).

As the pairing between the CS and the US was repeated over learning trials, the subsequent CS began to produce distinct ensemble patterns that were often not so obvious from the mere visual inspection of spike rasters (Fig. 2D). For example (as shown in Fig. 2E), by the third trial the conditioning tone triggered a robust dynamic trajectory, which started from the Rest ellipsoid, moved quickly to the conditioned Tone (CS) ellipsoid and then came back to the Rest ellipsoid.

More importantly, the foot shock (US) at this stage elicited the associative form of the ensemble trajectories that differed from the first US response: namely, the trajectory first visited the US cluster, but then shot directly to the CS cluster before returning to the Rest ellipsoid (Fig. 2F). This trajectory had the associative dynamic path that linked the foot shock ellipsoid to the conditioned tone ellipsoid. As a rule, this trial-dependent emergence of US-CS association traces (in response to the US) (Fig. 2G, also see Fig. S7C and S7D) was never present during the first US presentation (1^st^ pairing) across all recorded mice, but began to emerge during the second or third CS/US pairing (see group data in Fig. 2H, each yellow square indicates the presence of such associative trajectories). Of six recorded mice over seven learning trials, US-CS association patterns occurred 28 times out of a total 42 CS-paired US (7 trials x 6 mice), prevalent after multiple pairings (Fig. 2H). Thus, these associative trajectories, usually moving from the Rest-to-US-to-CS-to-Rest direction, reflect CA1 learning of the causal association of tone and foot shock.

### Hierarchical Organization of Response Selectivity within Neuron Population

Our observations of distinct ensemble encoding patterns during trace conditioning have prompted us to investigate how CA1 neurons correspond to various features of the events or stimuli. Thus, we utilized an agglomerative hierarchical clustering method to our data sets [28], [30]. The clustering analysis revealed the existence of various neural groups with distinct response properties (Fig. S6). Few cells (0.6% of the recorded units) seemed to be general, meaning that they responded to all types of stimuli [including CS before pairing (CS^b^), CS during the pairing (CS^d^), CS after the pairing (CS^a^), as well as US]. Some cells (6.5%) exhibited sub-general responsiveness (responding to CS^d^, CS^a^, and US); yet some cells changed their firing rates to CS^d^ and US (19.1%). Another set of cells responded to CS^a^ as well as US (1.7%). Interestingly, a small percentage of cells appeared to only respond to CS^b^ (2.2%), or CS^a^ (4.5%). We also observed that some CA1 cells were selective to the foot shock (18.2%) but not to the tones. Again, it is worthy note that variability of single neurons during the rest period and in response to stimuli (as shown in the spike rasters of Fig. S6A and S6C) creates a difficulty in predicting whether real-time memory traces were formed or retrieved in the hippocampus at a given moment within any single trial. Nonetheless, data averaging over trials (such as shown in Fig. S6B and S6D) provided useful information about the response-selectivity of these neurons. Overall, the response selectivity of CA1 cells can be classified in a hierarchical and categorical manner, ranging from general responsive units to various specific responsive units (Fig. S6E).

### Learning Pattern Replays during and between CS/US Pairings

**Figure 3:**
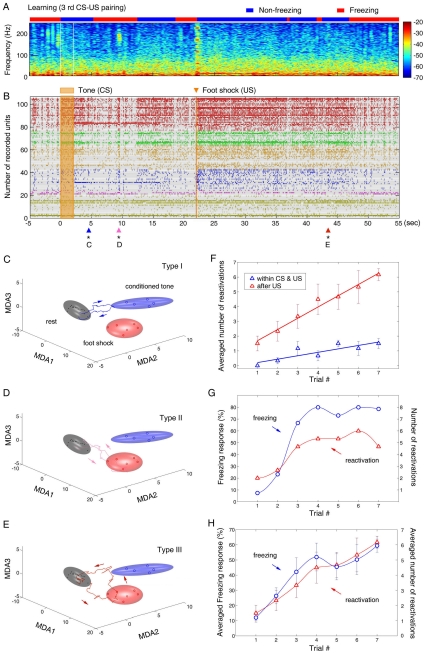
Reactivations of ensemble dynamic patterns during learning. (**A**) Spectrogram of local field potentials during the third CS-US pairing training. The blue color bar on the top indicates the non-freezing state, whereas the red bar indicates the freezing state of the animal. The color scale represents spectral power. The time scale is matched with the spike raster shown below. (**B**) Three post-stimulus immediate reactivations of the ensemble encoding patterns during the third learning trial were detected, marked by small triangles at the bottom of the spike raster. The sixty-sec spike-raster plot from mouse #1 is the same as the one shown in Fig. 2D. (**C**) An immediate reactivation, shown by the blue trajectory, was detected at the moment marked by the blue triangle at the bottom of (B). Similar to its original encoding pattern (shown in Fig. 2E), this reactivation trajectory transiently visited the CS cluster. This CS Trace (type-I) lasted about 0.8 seconds. (**D**) A second reactivation, shown by the pink trajectory, occurred at ∼9 sec shown by the pink triangle in (B). This trajectory visited the US cluster. This US Trace (Type-II) lasted about 0.7 seconds. (**E**) A third reactivation (red trajectory) took place at 43 sec, indicated by the red triangle in (B). This 1-sec association trajectory (Type-III), just like the encoding traces shown in Fig. 2F, first visited the US cluster and then toward the CS cluster before returning to the Rest. The same directionality was maintained. (**F**) Averaged numbers of reactivations (all three major types of traces) in six recorded mice over seven learning trials. The blue open triangles presents the averaged reactivation numbers within the 20-sec interval of the same CS-US pairing, whereas the red open triangles show the averaged reactivation numbers within the 60-sec after the paired US was delivered. The linear regression lines suggest the accumulative effects of pairings on the ensemble trace formation. (**G**) Numbers of immediate reactivations and the amounts of immediate freezing over seven trials in mouse #1. Cross-correlation analysis shows the correlation between freezing responses and occurrence numbers of reactivations was highly significant (r = 0.9657, p<0.001). Averaged freezing responses and the total numbers of reactivations were calculated from the period between the tone offset and the 60-sec after US presentation. (**H**) Averaged immediate freezing responses and the total numbers of reactivations were calculated from six mice. The correlation between freezing and reactivations was again highly significant (r = 0.9551, p<0.001).

At the behavioral level, the mice exhibited a significant amount of immediate freezing during training (seven pairings were given during a 10-min period). This behavioral freezing was accompanied by the prominent presence of significant ripple oscillations (see the spectrogram of local field potentials on the top panel of Fig. 3A). What is happening to the population-level encoding patterns during this period? To approach this question, we examined whether the ensemble encoding patterns triggered by CS/US stimuli would undergo spontaneous reactivations even during this learning phase. Our analysis revealed multiple spontaneous excursions from the Rest ellipsoid within the CS/US inter-stimuli interval as well as after the US presentation (sees triangles at the bottom of Fig. 3B). For example, during the third CS-US pairing, there were two reactivations occurring within the 20-sec CS-US interval. The first reactivated ensemble trace occurred at 2.8 seconds after the tone offset (the blue triangle at the 4.8-sec time point in Fig. 3B and 3C) and the second reactivated trace happened at 7.5 seconds after the tone offset (the pink triangle at the 9.5-sec time point in Fig. 3B and 3D) after the CS presentation ended. Also, one reactivation was further observed at 21.4 seconds after the offset of the US (the red triangle at the 43.4-sec time point in Fig. 3B and 3E). These post-stimulation, spontaneous trajectories (Fig. 3C, 3D and 3E) had the characteristic geometric shapes similar to the original ensemble traces (see Fig. 2E, 2C, and 2F). These spontaneous, transient trajectories also took place on a similar time-scale and exhibited the same directional specificity. Additionally, they were confined in the same plane as the original traces observed during the presentation of actual stimuli. Our measurement of the time points of these transient trajectories suggests that these immediate, spontaneous reactivations occurred very rapidly, typically within several seconds to minute(s) with random time intervals.

Importantly, we noticed that the averaged numbers of immediate reactivations (pooled from six recorded mice) within the 20-sec CS-US time interval increased in proportion to the numbers of conditioning trials (Fig. 3F, blue triangles and plot). Similarly, the numbers of reactivations that took place after the foot shock (during the 60-sec period after the paired foot shock) also increased over trials (Fig. 3F, red triangles and plots). Moreover, the increase in the total numbers of immediate reactivations was well correlated with the increase in the amounts of immediate freezing. For example, in mouse #1, both the total numbers of reactivations (all three major types) and the amounts of immediate freezing increased rapidly in the first three trials, but reached their plateaus by the fourth trial (Fig. 3G). Cross-correlation analysis showed that the correlation between freezing and reactivations was highly significant (r = 0.9657, p<0.001). Again, the group data (from six mice) also confirmed that the trial-dependent increases in the total numbers of reactivations (Fig. 3H) were highly correlated with the averaged amounts of immediate freezing over learning trials (r = 0.9551, p<0.001). Thus, the repeated CS/US pairings clearly had an accumulative effect on the formation/consolidation of various encoding patterns.

### Memory Traces during Contextual Fear Recall

**Figure 4:**
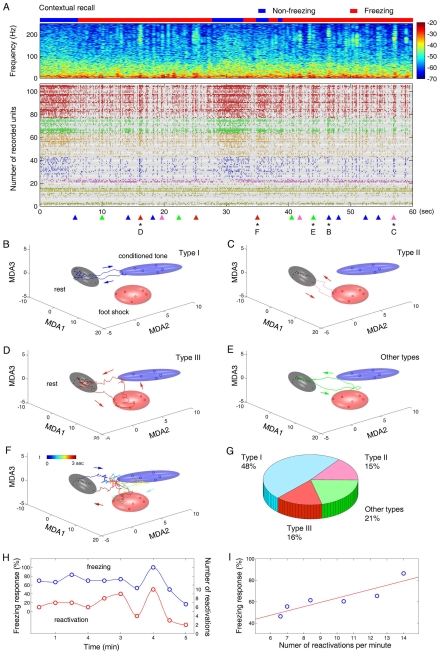
Ensemble dynamic patterns during contextual recall. (**A**) The top panel shows the spectrogram of local field potentials during the 60-sec epoch of the contextual recall test in mouse #1. The blue bar on the top indicates the non-freezing state, whereas the red bar indicates the freezing state of the animal. The color scale represents spectral power. The time scale is matched with the spike raster shown below. The colored triangles at the bottom indicate the moments at which encoding patterns re-appeared. (**B**) The blue trajectory shows that a representative CS trace (Type I) was retrieved, indicated by the star marked with B at the bottom of the raster in (A). This transient dynamic lasted about one second. (**C**) The pink trajectory shows a representative US trace that lasts about 0.7 seconds (marked by the pink triangle with C in (A), Type II). (**D**) The red trajectory is a representative US-CS association trace (the red triangle with D in (A), Type III). This trajectory occurred for about 0.8 seconds. (**E**) The green trace shows a type of different traces often seen during recall test. It visited the space between the US and CS clusters and lasted about one second. (**F**) The colored trajectory shows a Type III trace that was retrieved at the time indicated by the red triangle with F in (A). This trajectory occurred in the non-freezing state, and interestingly, had a reversed directionality, namely, moving from the CS ellipsoid to the US ellipsoid. It lasted about three seconds. (**G**) The percentages of different types of ensemble patterns during contextual recall in six mice. (**H**) Freezing responses and the total numbers of pattern retrievals were calculated and compared during the entire 5-min contextual recall test (mouse #1). The blue circles are the freezing responses counted in every 30 seconds; the red circles show the averaged numbers of three major types of ensemble traces counted in the same 30 seconds. Cross-correlation analysis shows that the correlation between freezing responses and occurrences of pattern retrievals was significant (r = 0.8840, p<0.001). (**I**) Averaged freezing responses in six mice are also tightly correlated with their averaged numbers of total pattern retrievals during the contextual recall tests (r = 0.8956, p<0.05). Each circle represents the data from a single mouse. This between-animals plot indicates that the numbers of pattern retrieved is almost in linear proportion to behavioral performances as measured by the amounts of contextual freezing. The animal which had a fewer number of pattern retrieved also showed lower fear memory freezing, whereas the mice which had high numbers of pattern recalled exhibited highest amount of freezing.

During recall tests, we first measured contextual fear memory using a one-hour contextual retention test which engages both the hippocampus and amygdala [8], [18]–[21], [31], [32]. As expected, the conditioned mice exhibited robust freezing when the animals were re-exposed to the original shock chamber (Fig. 1E and 1G). Our analysis revealed that the re-exposure of the animals to these contextual cues triggered rapid and repeated re-emergence of the various encoding ensemble patterns, often in tandem during the freezing epoch (Fig. 4A). Importantly, the appearance of the first CA1 recall pattern always preceded the onset of the first behavioral freezing, with an averaged leading time of about 1.4 seconds. This fits well with the predicted causal relationship between fear memory trace recall and behavioral readout.

For example, mouse #1 entered the freezing state 8 seconds after re-introduced to the conditioning chamber. The observed first retrieved pattern emerged 360 mini-seconds before the animal froze (see the top red bar in Fig. 4A). During the first freezing epoch (∼23 seconds), eight transient trajectories were detected (see triangles at the bottom of Fig. 4A). These dynamic ensemble trajectories oscillated among the Rest-to-CS cluster (Fig. 4B, Type-I), Rest-to-US cluster (Fig. 4C, Type-II), and Rest-to-US-CS association subspace (Fig. 4D, Type-III). In the second freezing epoch that lasted about 20 seconds, another set of trace retrievals (8 trajectories) was observed (see triangles in Fig. 4A bottom, right side). The order of the appearances of these ensemble traces during the second freezing epoch was different from that during the first epoch.

In addition to the three major types of ensemble trajectories that were originally seen during the learning phase, we also noticed that other types of trajectories were present during contextual recall. Some trajectories would fall between the CS and the US ellipsoids without directly touching either one of them (Fig. 4E, other types). Interestingly, we also observed that on several occasions a new kind of US-CS association trajectories appeared. These trajectories showed a reversed movement (CS-to-US), moving from the Rest to the CS cluster and then directly to US cluster before returning to the Rest (Fig. 4F). Our composition analysis of the pooled data from six mice shows that 48% of the various traces retrieved belonged to Type-I (CS-traces); 15% and 16% of the retrieved patterns were Type-II (US traces) and Type-III (CS-US association traces), respectively; while other types constituted the remaining 21% (Fig. 4G).

Interestingly, the numbers of ensemble patterns retrieved were also highly correlated with the amounts of freezing during contextual recall. As illustrated in Fig. 4H, the numbers of patterns recalled in mouse #1 co-varied tightly with the amounts of behavioral freezing over the 5-min period of the contextual recall test. The fitting curves showed a highly significant correlation between the freezing response and the occurrence of the retrieved ensemble traces (r = 0.8840, p<0.001). Furthermore, the averaged numbers of memory patterns retrieved during contextual recall in six recorded mice also exhibited a near linear function over averaged freezing responses (Fig. 4I, r = 0.8956, p<0.05).

During contextual freezing, we noted that there was a dramatic increase in the number of sharp-wave ripples, which can be detected by filtering and thresholding the CA1 local field potentials in the 100∼250 Hz (ripple) frequency band (Fig. S8A and S8B). As evident from the power spectral density plot (Fig. S8B), the ripples were prominently taking place during freezing, but not in the non-freezing state of the recall sessions. Since ripples have been shown to increase after learning behaviors in rats [33]–[36], we investigated the relationship between ensemble trace retrievals and ripple occurrences. We measured the exact time points for both ensemble pattern retrievals and hippocampal ripples and then compared the co-occurrences of these two types of the events (Fig. S8C-F). Our analysis shows that 87.5% of ripples occurred during the contextual freezing state were associated with ensemble pattern retrievals (±1 sec time window) (Fig. S8G). Conversely, 49.2% of ensemble pattern retrievals were accompanied (within ±1 sec time window) with obvious ripples (5 standard deviation above the mean power) (Fig. S8H).

### Memory Patterns during Traced-Fear Recall

**Figure 5:**
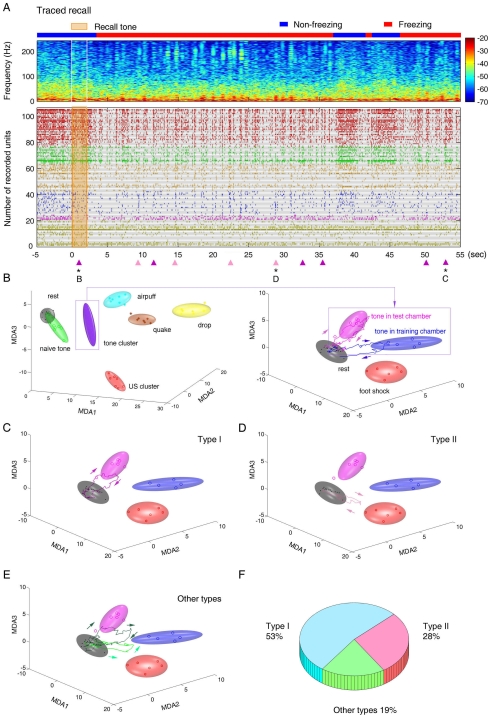
Ensemble dynamic patterns during the trace recall test. (**A**) The top panel shows the spectrogram of local field potentials during a 60-sec epoch of trace recall in mouse #1. The blue bar on the top indicates the non-freezing state, whereas the red bar indicates the freezing state of the animal. The time scale is matched with the spike raster shown below. Colored triangles at the bottom indicate the various moments at which encoding patterns were retrieved. To initiate the trace memory recall, a 2-sec tone was played. (**B**) Global MDA analysis shows that the data from the recall chamber and the conditioning chamber formed a single tone-pattern ellipsoid. The conditioned tone patterns (see the rectangle in the left panel) were recomputed by a second-step MDA to achieve finer separation for the difference of contextual environments. The purple trajectory is the recall-triggered CS pattern at the time when the tone was played, indicated by the purple triangle with a starred B in (A). (**C**) Type-I trajectory (purple) was retrieved during the first 1-min trace recall trial. This trajectory occurred at 53.5 seconds, indicated by the purple triangle with a starred C in (A). (**D**) Type-II trajectory (pink) was retrieved during trace recall. This trajectory occurred at 28.2 seconds, indicated by the pink triangle with a starred D in (A). (**E**) Additional types of ensemble trajectories were also detected during the trace recall trials. In particular, the dark green trajectory seemed to visit both the tone-at-recall cluster and the tone-at-training cluster. Whereas the light green trajectory visited the subspace between the tone-at-training cluster and the US cluster. (**F**) The averaged percentages of different types of ensemble patterns retrieved during the trace recall sessions from six recorded mice.

After the contextual retention test, the mice were subjected to the one-hour trace memory recall test by placing the animals in a non-conditioned chamber which had a different floor, color, context, and geometric shape (see Fig. 1A). In our trace retention test, the tone was given seven times with a 1-min interval between each presentation. As expected, prior to the re-exposure to the recall tone, the mice had a very low amount of freezing (Fig. 1F and 1H; see the top blue bar Fig. 5A). However, upon the presentation of the recall cue, the animals quickly entered the freezing state (Fig. 5A, red bar, also see Fig. 1F and 1H). The behavioral measurement shows that the time latency between the end of the 2-sec tone presentation and the onset of cued freezing was between 0.5 to 5 seconds (averaged at 1.98±1.51 sec) (see Fig. S9).

Since the tone during trace recall was presented to the animals in a novel environment which was different from the original training chamber, we first examined whether the major information carried by conditioned tones would change as the contextual environments differed. We approached this question by assessing various categories of distinct events in the global MDA analysis. Our analysis shows that tone-triggered responses during both learning and the recall test could still form a single CS cluster that was well separated from all other events (Fig. 5B, left panel). This indicates that the major information carried by the tones during training and the recall was similar despite the contextual difference. However, the subtle contextual difference could be resolved by using a second-step MDA analysis under which the CS ensemble trajectories elicited during recall can be separated from the CS ensemble trajectories during training (Fig. 5B, blue vs. pink ellipsoids in the right panel).

With this sensitive 2-step MDA method, we assessed how ensemble patterns were retrieved during trace recall. We found that the cue (tone) triggered a string of ensemble pattern retrievals (9 transient trajectories) within the 1-min period (see various colored triangles at the bottom of Fig. 5A). These dynamic trajectories included tone traces (Fig. 5C) and shock traces (Fig. 5D), as well as other types of traces (Fig. 5E; for additional examples, see Fig. S10). Our statistical analysis of major trajectories retrieved from the six mice showed that the conditioned tone patterns constituted about 53% of the total numbers of pattern retrieval, the shock patterns were about 28%, and the remaining 19% belonged to other types.

**Figure 6:**
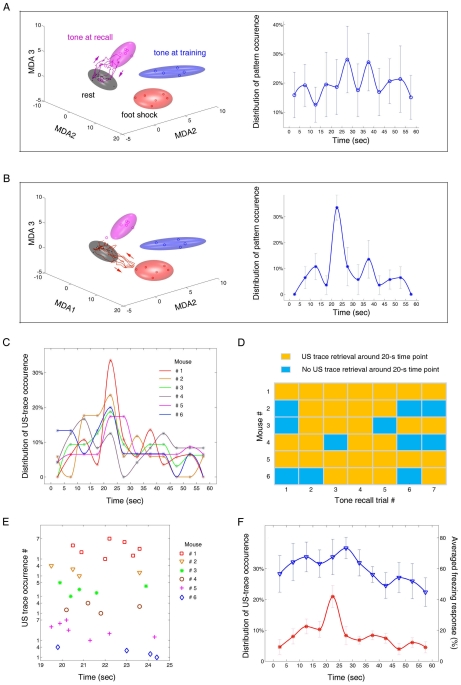
The peaked re-appearance of the US patterns at the traced time interval indicates the memory of time. (**A**) Representative ensemble tone traces during trace recall (Left panel). The right panel shows the time distribution of the averaged occurrences of this type of trajectories over seven recall-trials in mouse #1. Time zero indicates the moment when the recall tone was delivered. The numbers of trajectories were pooled every 5 seconds. (**B**) Representative ensemble shock traces during the traced memory recall session are shown (Left panel). The right panel shows the time distribution of the averaged occurrences of this type of trajectories over seven recall-trials in the same mouse. The same analysis reveals a salient peak in the occurrences of the US trajectories around the traced interval time point. Time zero indicates the onset moment when the recall tone was delivered. The tone lasted 2 seconds, thus the absolute amount of time between the offset of the tone and the retrieval peak was around 20 seconds. (**C**) The peaked occurrences of the US ensemble trajectories were observed in five out of the six recorded mice. Each colored plot represents a mouse. (**D**) The color matrix shows the retrievals of the US ensemble traces in six individual mice around the traced interval time (20±2.5 sec after the offset of the recall tone) at each of the seven trials. Yellow squares represent the occurrences of the correct US ensemble patterns, whereas blue squares indicate the absence of US patterns at the time point of this trace interval. (**E**) The exact time distribution of the 31 correct US pattern retrievals around the traced interval time in all six mice. Please note that the 22-sec time point is from the onset of the recall tone (which lasts 2 seconds). (**F**) Lack of distinct peak in the traced fear freezing over the 1-min recall trials. Blue triangles show the five-second averaged freezing responses over seven recall-trials (1-min per trial) from six recorded mice. Red stars show the US pattern retrieval distribution. Correlation between the averaged freezing response and occurrences of US traces was still significant (r = 0.5851, p<0.05), despite the absence of a distinct peak in freezing.

To further understand the temporal dynamics of trace memory recall, we plotted the time distribution of various ensemble trace retrievals over the retention test period. We found that the conditioned tone patterns underwent multiple retrievals in mouse #1, fluctuating over each 1-min recall session (Fig. 6A). Interestingly, the ensemble traces specifically corresponding to the shock experiences exhibited a distinct recall peak around 20 seconds after the offset of the tone, which is the expected moment for the shock (Fig. 6B). The peak retrievals of the US ensemble traces around the 20-sec time point were observed in five out of six recorded mice (Fig. 6C). On the trial-to-trial basis, the US ensemble patterns consistently re-appeared around this moment in all seven recall trials for mouse #1 and #5; whereas the other four mice had the US trace retrieval at this right moment four or five times out of the total seven trials (Fig. 6D). Collectively, out of 42 tone-trace recall trials, 31 US-trace retrievals were observed in these mice. The exact time distribution of US retrievals around this 20-sec time point in these mice was shown in Fig. 6F (22-seconds after the onset of the 2-sec tone). The fact that it was the US ensemble pattern being uniquely and consistently re-elicited at this conditioned time interval suggests that these ensemble trajectories further signifies the retention of memory for time. It is noteworthy to point out that there was no salient peak in behavioral freezing during this 1-min trace recall period (Fig. 6F). However, there is a still significant, but lower, correlation between the reactivation numbers and the amounts of freezing (r = 0.5851, p<0.05). The lack of significant changes in freezing behavior nicely illustrates that unprecedented decoding power of the large-scale recording and decoding algorithms. This is especially valuable given the fact that various memory traces, including cued fear memory, can all cause freezing upon the tone-recall, which is exceedingly difficult to be distinguished by measuring freezing behaviors.

## Discussion

Extensive behavioral and lesion studies show that the hippocampus is involved in both the encoding and retrieval of various associative memories, including trace fear conditioning memory [3], [4], [6], [7], [18]–[23]. By the classical definition, real-time memory traces are the neural population-level dynamic patterns that are initially formed during learning and would then reappear at the time when animals actively engage in cue-induced memory recall. Neuroscientists try to decipher the brain's memory traces by searching for reliable correlation between firing patterns of neurons and behavioral measurement. However, due to a large amount of response-variability at the single neuron level in the brain even in response to identical stimulus, single neuron-based decoding schemes often produce significant errors in predictions about the stimulus identities or external and internal information. The traditional way to deal with the response-variability of single neurons is to average spike discharge of the neurons over repeated trials. Although the data averaging across trials permits the identification of tuning properties of the individual neurons, unfortunately, this practice invariably loses crucial information regarding real-time encoding process in the brain. In fact, the brain is unlikely to use the data averaging over many trials as the way to represent snap-shot memories because a memory is a transient state at a given moment in time. Moreover, in many situations, there is no explicit time point to be used as time zero for averaging spike raster. A good example is that in the contextual fear retention test, there is no fixed time point for the animals to recall fear memory after they re-enter the shock chamber. In addition, the traditional peri-event spike raster or histogram methods have limited the analysis to a single time point when the tone or cue was presented at retention tests (e.g. the time point when the tone is played as time zero for peri-event plots in the single neuron approach). But this would overlook other subsequent memory traces that might appear in tandem with variable intervals between each single trace. As a result, the use of recall-tone as time zero would average out such valuable information.

On the other hand, many laboratories have focused on place cell models to study hippocampal activity patterns. Despite of enormous information has been collected over the past several decades, there are on-going controversies within the place cell researchers as to whether place cell phenomenon qualifies as episodic memory phenomenon or merely reflects some kinds of sensory responses during spatial path integration [37], [38]. Intriguingly, it has been kept within the place cell research community that place cells loss their firing selectivity if the animals are not running at or above a certain speed (e.g. if a rat sits right at the place field of a given place cell, that particular place cell would not exhibit much place-selectivity, but rather fluctuate its firing widely) [39]. This peculiar dependency of place-selective firing on running motion seems to differ from our ability in forming or recalling spatial memory of places (which can occur regardless we are in motion or sit still). This raises the key question as to what degree place firing reflects passive sensory responses as to actual memory encoding.

Moreover, the dilemma of place cell studies as an episodic or associative memory paradigm may also lie at the difficulty in using this model for classic associative conditioning (due to the continuous variables during spatial running). As a result, the direct link between place cell firings and behavioral readout in memory acquisition and retention tests has not been demonstrated so far. For example, this is no evidence in explicitly linking place cell replay to cue-induced behavioral memory readout at memory retention tests (e.g. to demonstrate that place cell firing would replay upon presenting a recall cue with the rats not in running motion, and then to correlate such replay with behavioral scores of spatial memories).

To overcome the above technical or experimental limitations, the present study employs the classic Pavlov fear conditioning paradigm, coupled with large-scale recording and decoding methods to investigate the real-time population-level activity patterns in the hippocampus in relation with trial-to-trial memory scores. The choice of Pavlov conditioning offers several advantages: 1) the hippocampus is widely known to be crucial for such associative function, especially with longer CS-US interval and context; 2) the well defined cues allow researchers to measure the behavioral readout for memory formation and then to correlate behavioral readout with transient ensemble patterns, thereby fulfilling the definition for the observed transient dynamics as actual memory traces.

What do real-time memory traces in the hippocampus look like over the course of learning and recall? How do the associative memory traces differ from unconditioned stimulus-evoked responses? Does the brain retrieve a single memory trace or multiple memory traces upon a cued signal? If a train of memory traces are retrieved, what are they? And are there any temporal structures or relationship in its recall sequence? How do memory traces correlate with behavioral performances during the recall tests?

These fundamental questions are fascinating, and the trace conditioning task is an ideal paradigm to examine these issues since it is well known to produce a variety of memories (at least in the human brain) that can include a shock or air-puff event, the causal and temporal relationship between a tone and a shock, as well as the environment in which an event occurs. It is conceivable that rich collection of various memory components can all contribute to and/or influence behavioral outputs.

It is often acknowledged that behavioral readout represents a good, but not very precise, assessment of the internal memory processes in the brain. By using large-scale ensemble recording techniques and statistical pattern recognition algorithms [17], [28], [40], we systematically assessed and scanned the recorded neural activity patterns during the acquisition and retrieval of this classic associative memory. Obviously, merely visual inspection of the spike rasters are not very effective to detect and understand the neural population-level patterns and dynamics in the high dimensional data, we have employed MDA method capable of measuring and visualizing the ensemble neural activity patterns. Because our sliding window is 10 milli-seconds, this means that continuous fine points showed for trajectories in MDA space represent the activity changes in 10 ms time resolution. This sliding window scans the entire recording session on the moment-to-moment basis, thereby providing unprecedented insights into how the hippocampus encodes and retrieves memory. It is worthy note to point out that 500 ms time epoch for MDA calculation was chosen based on the fact that most CA1 neurons' responses occurred in this time range, thus this time bin can best capture such features. Coupling of these matrix features with the 10 ms-high temporal resolution sliding window has enabled us to look into the real-time transient dynamics associated with memory acquisition and recall in the following ways:

First, CA1 ensemble patterns evoked by the CS-US pairing change rapidly from unconditioned forms to conditioned associative forms. That is, the CS-US pairing resulted in the rapid appearance of robust CS ensemble traces after one or two trials in the mouse CA1 region. Interestingly, as the conditioned tone trace emerged, US-triggered ensemble responses which originally evoked only US-specific transient trajectories began to turn into the US-to-CS association trajectories. The emergence of these new types of association trajectories suggests that circuitry-level dynamics have succeeded in capturing the CS-US causal relationship. Interestingly, the temporal sequence of association trace is reflected by its movement direction: these transient trajectories moved from the US cluster to the CS cluster in the MDA encoding subspaces. These associative patterns required the repetition of CS/US pairings, and can appear as early as the second pairing and become prevalent during the late stage of learning phase in all animals. The formation of such associative memory traces is likely the product of synaptic plasticity. It would be of great interest to examine the molecular basis of these associative patterns in using NMDA receptor conditional knockouts or other genetically modified animals with enhanced or impaired memory or in which memory can be rapidly erased [41]–[44].

Second, the encoding patterns produced by learning replay immediately within seconds during the trace conditioning acquisition phase, and more importantly, they correlated tightly with learning scores. The reappearance of temporal trajectories often retained their original directional movement and geometric shapes and subspace plane. Importantly, the immediate spontaneous reactivations following CS/US stimulations exhibited a clear trial-dependent property, that is, the numbers of reactivations increase in proportion to trial numbers. While there are few reactivations at the beginning of the trials (e.g. the first pairing), reactivations become more prevalent at late trials. The near linear correlation between the trace reactivation numbers and the levels of behavioral learning (immediate freezing) suggests that post-learning immediate reactivation may be an important physiological indicator for assessing network-level trace formation in the hippocampus. Our finding collaborates well with the previous findings showing that neurons in the brain also undergo reactivations after spatial running [36], [45]–[47] or novelty stimulation [48] or episodic events [17], [28]. Importantly, our present study has greatly extended those observations by linking the pattern replay frequency with behavioral scores of learning.

Third, to meet the primary criterion that transient neural ensemble patterns represent true memory traces, one need to demonstrate that those patterns observed during learning are to be retrieved upon the recall cues and they would again correlate with memory scores. In line of this criterion, we have indeed observed that these CA1 dynamics patterns reappeared at the time of both contextual and traced fear retention tests. Using contextual cues or trace cue (tone) in the absence of shock is important feature for demonstrating classic associative memories. Importantly, this well defined, retention test has offered us to link the physiological ensemble patterns to cued-induced memory recall performances that so far have not been demonstrated in place cell model experiments [37].

We find that on average, ensemble traces were retrieved at a rate of 6-14 times per minute in the mouse hippocampus during the retention tests. Importantly, the numbers of retrieved patterns in the context recall tests were tightly correlated with behavioral performances in the memory retention tests at both the individual and group levels, thereby meeting the criteria of functional readout.

It is also important to note that the time scale for retrieved memory patterns in our recall test is similar to that of the memory encoding patterns in the learning phase. Furthermore, as predicted, we have observed the causal relationship between appearances of the first memory trace and the recall-induced freezing. That is, the first recalled memory trace consistently precedes freezing behavior on the average of 1.4 seconds. Given the fact that we calculated the time delay between the off-set of the first memory trace and the onset of freezing, the causality should be beyond questionable. Thus, our present experiments provide a piece of crucial evidence that neural ensemble patterns formed during learning indeed reappear and precede behavioral recall, thereby fulfilling the major criterion for those transient dynamics as population-level memory traces.

Intriguingly, some ensemble traces or trajectories observed during recall were not seen during the learning phase (e.g. the reversal of CS-to-US association trajectory sequence rather than the original US-to-CS association trajectory movement). Emergence of richer ensemble patterns at the time of recall may reflect not only the successful retention of various associative relationships at the time of learning but also the possible on-going re-modification or updating of existing memories by the recall experience itself [8], [23], [24], [49]–[55]. At this stage, we do not know whether this reversal trajectory movement reflects the fear extinction process as the tone was played without reinforcement of shock pairing.

Fourth, the observation that the significant amounts of ensemble pattern retrievals were accompanied by sharp-wave ripples provides us a unique opportunity to examine the correlation of memory processing with hippocampal ripples that are often reported in the literature [33]–[35], [56]. Our analysis indicates that near half (about 49%) of ensemble pattern retrievals were accompanied (within ±1 sec time window) with obvious ripples, whereas the other half were not. It is possible that remaining recall patterns in the absence of obvious ripples may be still accompanied by subtle ripples that were less than 5 standard-deviations above the mean power). An alternative interpretation would be that hippocampal ripples play an important, but not necessarily essential role in memory recall. This may explain well why the effects of suppression of ripples on spatial memory were observable, but relatively mild [56]. In the fear conditioning paradigm, it is likely that CA1 ripples might reflect the effect of behavioral immobility on hippocampal oscillatory properties because ripples are known to occur uniquely during the state of locomotion immobility in rodents. Future studies will be needed to clarify this issue.

Fifth, our decoding method has revealed the memory of time for the expected shock events. The unique retrieval peak for the US shock traces at the time point of expected shock suggests that the animals retained not only the memory of the correct event, but also the memory of its timing. This level of decoding power is particularly interesting to us since at the behavioral level the animals were in the heightened freezing state through the most of the recall time (at the scale of 1-minute). This persistent freezing reduces the sensitivity of behavioral readout, and thereby, unable to allow the distinction of various memory components (contextual memory, cued memory, time memory, etc). For example, the presentation of a recall tone would easily cause the animals to freeze right away because the mice retrieved tone/shock association memories, this can easily mask the behavioral readout for the memory for time for the expected shock event. This level of decoding power is highly useful given the fact that many of our own memory thought processes can occur without necessarily being expressed at behavioral level intentionally or unintentionally.

From our analyses, it is quite clear that CA1 ensemble traces that had undergone trace memory recall are rich and diverse. Quite often, the various ensemble trajectories were retrieved in tandem (∼9–10 times per minute on average during either contextual recall or trace recall). Although our visual inspection has not revealed any obvious simple-order temporal sequences in their emergences, it remains possible that some types of high-order temporal relationship may still exist. On the other hand, cognitive literatures often emphasize that memory contents/or sequences seem to always differ slightly at each recall, even when the human subjects were presented with the same question. Thus, it would be a great topic for more detailed investigations.

It has been known that many brain regions are engaged in processing associative memories [5]–[7], [31], [32], [57]–[62], so it is essential to understand the ensemble patterns in these regions during trace fear conditioning in future experiments. Moreover, since our trace conditioning protocol requires animals to hold their attention for long-period of time (20 seconds), it will be logical to explore the roles of attention and working memory component on hippocampal memory representation [57], [63], [64]. The time duration of CS-US interval is an important factor for trace memory in trace fear conditioning, so one may also study how the interval influences the accuracy of the memory for time. All together, it is likely that the investigations of the above questions will lead to better understanding of how the hippocampal ensemble patterns described here contribute to the global representation of various episodic memories in the brain [65].

In conclusion, the population-level associative memory patterns in real-time can be mathematically described and intuitively visualized on a moment-to-moment basis at any given single trial. More importantly, our present study demonstrates the meaningful relationship of these transient neural dynamics of memory traces with behavioral scores of learning and recall not only within each animal but also across animals. The ability to decode brain's diverse memory traces in real-time from the acquisition to recall phase may have wide implications and many useful applications.

## Methods

### Ethic Statements

All animal work described in the study have been conducted according to NIH guidelines and approved by MCG Institutional IACUC committee.

### In Vivo Recording and Spike Sorting

96-channel or 128-channel recording arrays were constructed as previously described [27], [28] and employed to record neural activity from hippocampal CA1 region in freely behaving mice (ten male B6BCA/J mice, six mice with the best unit yield were analyzed here). Each steretrode/tetrode was constructed by twisting a folded piece of 25/13 µm H-Formvar wire and was thread through one of the polyimide tubes. The spacing of our steretrodes/tetrodes is about 50–150 µm. Each mouse was implanted with two independently movable bundles of either 32 steretrodes or 16 tetrodes (64 channels on each side of the hippocami) to bilateral hippocampi under deep anesthesia using 60 mg/kg ketamine (Bedford Laboratories, OH) and 4 mg/kg Dormitor (Pfizer Animal Health, NY). The electrode bundles were positioned above the dorsal hippocampi (2.0 mm lateral to bregma and 2.3 posterior to bregma on both right and left sides). After the mice recovered from surgery, the electrodes were advanced slowly, over next five to ten days, through the cortex in daily increments of about 0.07 mm until the tips of the electrodes reached the pyramidal layers of the hippocampal CA1 region. The electrodes were formatted in either stereotrodes or tetrodes.

The spike activity was recorded using Plexon Systems (Dallas) and then sorted using the MClust 3.3 program (http://www.cbc.umn.edu/~redish/mclust, David Redish). First, the recorded data were filed as Plexon system format (*.plx). Before spike sorting, the artifact waveforms were removed and the spike waveform minima were aligned using the Offline Sorter 2.8 software (http://www.plexon.com, Dallas, TX). The aligned data were then saved as files in Neuralynx system format (*.nst). After that, the MClust 3.3 program was used to isolate different spiking units. Only units with clear boundaries and less than 0.5% of spike intervals within a 1 ms refractory period are included in the present analysis.

Six sets of recording data were obtained from six mice for the current analysis. The number of each recorded dataset contained 208 sorted units (for mouse#1), 258 units (for mouse#2), 242 units (for mouse#3), 232 units (for mouse#4), 308 units (for mouse#5), and 206 units (mouse#6), respectively. Isolated units were classified as either putative excitatory pyramidal cells or interneurons based on their characteristic firing activity including waveforms, inter-spike intervals, and firing rates [66]. In general, pyramidal cells fire at lower rates (<5 Hz) and have broader waveforms (>300 µs); whereas, interneurons show the higher rates (>5 Hz) and have relatively narrower waveforms. Additionally, Pyramidal cells occasionally fire complex-spike bursts of two to seven spikes at 3–10 ms inter-spike intervals, reflected by their characteristic autocorrelograms and inter-spike interval histograms. Further analysis is based on the sorted data; therefore, no electric artifacts were included during the shock events (Fig. S3, S4). To confirm the recording sites of the electrodes, all recorded animals were anesthetized after all the experiments and 10 µA current was applied to each recording electrode for 5 seconds in order to mark the positions of the stereotrode bundles. Nissl staining was used to confirm the electrode positions. The stability of the ensemble recordings were judged by waveforms and inter-spike-intervals at the beginning, during, and after the experiments. Some of the representative units were shown in Fig. S2 and S3 (including examples from stereotrodes and tetrodes).

### Fear Conditioning Task

The fear conditioning chamber was a square chamber (10″×10″×15″) made of plastic boards with a 24-bar inescapable shock grid floor, and the recall chamber was a triangular chamber made of foam boards with a smooth and opaque floor. Thus, these two chambers are distinct geometrically, contextually, and visually. Both chambers had one transparent wall, and thus freezing responses of animals in the chamber could be observed by experimenters from the small hole on the surrounding black curtain and were automatically videotaped by computers. In each test, only one animal was trained and recorded. Before training, the mouse was habituated in both chambers for five minutes per day, and three days in total (a tone was played 10 times when the mice were habituated in contextual chamber). We and others have found that some CA1 cells may respond to novelty (such as a tone that the animals have never heard before). But the neutral tone triggered responses tend to decline dramatically. The habituation protocol is used widely in the field because it gives the animals time to better differentiate CS tone with other sound or noise (thereby, reducing sound generalization).

On the training day, the recording began with a 1-h pre-training sleep period in the home cage (a plastic tub where the mice lived everyday) and then followed by a 3-min pre-training exploration period in the shock chamber and then a 30-min period during which a 2-sec tone (85 dB continuous tonic sound at 2800 Hz) was played ten times at random intervals. This allowed us to determine the CA1 responses to naïve tone. The animal was then brought back to the home cage for a 30-min break before trace fear conditioning began. During trace conditioning, the conditioned stimulus (tone, 2-sec, 85 dB continuous tonic sound at 2800 Hz) was given and then followed by a 20-sec interval (after the off-set of the tone) before the unconditioned stimulus (a continuous 285-ms foot shock at 0.75 mA) was delivered in the shock chamber. This CS-US pairing was repeated for seven times, with 1–3 min random time intervals between CS/US sessions. The mouse was then brought back to the home cage for 1-h post-training sleep/rest. The immediate freezing was assessed for 30 seconds after each shock.

After a one-hour rest, two kinds of memory retention/recall tests were conducted. The first one was the contextual memory recall test during which the mouse was placed back to the shock chamber again for five minutes. The other retention test was the trace memory recall test during which the mouse was put into a novel chamber and allowed to explore freely for three minutes before the onset of a 2-sec tone was played (repeated seven times with a fixed 1-min interval). After the completion of all retention tests, the mouse was returned to its home cage to rest for 30 minutes. After the rest, the animal was then subjected to four additional episodic events including metal sound, air-puff (10 p.s.i, 200 ms), earthquake-like shake (300 rpm, 200 ms), and drop (from the 13 cm height). The last set of episodic events was conducted after one-hour fear memory tests have been completed, and thereby would not affect data on fear conditioning. Inclusion of these startling episodic events was designed to provide additional information when we performed MDA analysis (e.g. allowing us to assess the global classification patterns among various stimuli and further define some of the fine details). Such information would permit us to ensure the statistical pattern classifications to be highly reliable in multiple MDA subspaces.

Throughout all these procedures, animal behaviors were recorded simultaneously by videotapes and synchronized with spike data collection. Freezing response, defined as absence of body movement except for respiration, was counted as the measurement of fear memory. Immediate freezing responses during training were measured for 60 seconds after the foot shock. We counted ‘freezing’ by observing the animal behaviors frame by frame based on the recorded video, the temporal resolution for the video is 30 frames per second and the absolute amount of time that the mice stayed in the continuous frozen state was summed and compared to the total amount of time during which the mice were not in the non-frozen state. The behavioral data presented in Fig. 1D–H were generated from ten mice, with the remaining figures and data collected from six of these ten mice. We used computer-controlled mechanical devices for controlling the precise timing and intensity of various stimuli.

### Data Processing and Projection Method Analysis

A 500-ms bin was selected for sampling ensemble firing activity based on the optimization of achieving the best statistical prediction power [28], [30]. Firing frequencies (*f*) were evaluated in two 500-ms time bins immediately after the events started. Neuron responses were defined by: 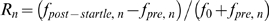 where *f*_0_ (2–3 Hz) is the global mean response frequency of putative excitatory neurons across the recorded neural population in an animal, excluding high firing-rate interneurons (cut-off at 20 Hz) and f_pre, n_ is the base firing rate (computed from samples of firing rates in two 500-ms time bins before startle stimuli). This transformation emphasizes significant changes in firing patterns for units with both low- and high baseline firing rates. Effectively, responses of low-firing units are proportional to absolute firing rate changes, while responses of high-baseline units are proportional to the relative changes.

We used Multiple Discriminant Analysis (MDA) projection method to classify and separate the neural responses corresponding to different episodes into different classes [28], [30]; these methods generate an encoding subspace of low dimension (on the order of number of classes). The projection method is useful in revealing the inherent hierarchical structure that may exist in large-size neural populations. Briefly, we computed firing frequencies (f) in two 500-ms time bins immediately after the delivery of the stimuli. Baseline activities were characterized by computing the average firing rates during time intervals preceding the startle stimuli. The selection of 0.5 sec for bin size for MDA analysis is not arbitrary, but rather based on the optimization for achieving the best statistical prediction power [28], [30]. We have described in great detail about how to best capture the statistical information from large neural datasets using various multi-variant methods [30]. In general, the selection of the input data can have a profound impact on the performances of the statistical methods. For example, while the duration of hippocampal neural responses to tone or shock ranges from a few hundred milliseconds to tens of seconds, the majority of such responses are within one second (but larger than 50 or 100 ms). As such, a selection of too narrow a time-window (such as 10 ms) would not capture critical details of these responses, as it would cut off a large part of the relevant spike responses. On the other hand, selection of a time window (such as 10 seconds) would be too large because the majority of the neurons have returned to the base firing rates after a few seconds [30]. Also, previous research suggests that multivariate methods perform better when the bin sizes used to create the input data sets have a width above 100 ms in primary sensory and motor regions. For our data sets we chose a 500 msec time interval to include information about the initial activation and the subsequent sustained activity, because the time bins are large enough to be robust to time variation that may be caused by delays in responses after the stimuli. A more detailed partition, of three or more bins in the 1 second interval could improve the classification performances, however as the dimension of the original subspace grows, the computation becomes increasingly under-determined. For our experimental data, we found that using time bins with widths between 250 and 500 ms yield similar optimal performances for the test data sets prediction. For more information, we have explored this issue in detail and have further compared it systematically with several statistical projection methods [30].

One set of population activity from seven repetitions of each type of stimuli was randomly chosen to constitute our test data set. The rest of the sampled population activities were then used to train our MDA statistical model. The matrix of mean responses during each category (rest and startle states) was then computed and used to compute the between-class scatter matrix 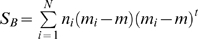: Here **n_i_** is the number of elements in each class, **N** is the number of classes, **m_i_** is the mean vector for each class and **m** is the global mean vector. To take into account the variations occurring for each class, we also computed the within-class scatter matrix **S_W_**, which is defined as: 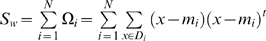. Here **D_i_** represents the set of population responses triggered by the **i** ^th^ startle type. Using these two matrices, it follows that a set of at most **N-1** discriminant projection vectors can be determined by computing the eigenvalue decomposition of the matrix 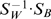.

For our data sets, the class covariance matrices **S_W_** are non-invertible, which is a direct consequence of data under-sampling, since the dimension of the number of recorded neurons is much higher than the number of repeated trials. In practice, the matrix **S_W_** can be rendered invertible using a regularization technique which changes each class covariance matrices based on the following formula: 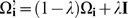, where 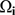 is the covariance matrix for the **i** ^th^ class, **λ** is a regularization parameter between 0 and 1, and **I** is the identity matrix. The parameter **λ** is determined automatically for each data set based on the optimization procedure we developed previously; each particular choice is determined by the particular distributions within each data set [30]. After the computation of **N – 1** discriminant dimensions, we projected the neural patterns during startle episodes in the low-dimensional encoding subspaces. We then used the multivariate Gaussian distribution probability functions (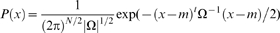) to fit the projections for each class. We subsequently enhanced our intuition about the relationships among classes by visualizing the 2σ boundary ellipsoids for each class. We tested the robustness of our MDA statistical model by employing different partitions of the training and test data points. The use of the Multiple Discriminant Analysis technique indicates that all different types of stimulations can be successfully classified. In addition, we used a sliding-window method on 10-ms to 10-ms basis to monitor the evolution of the population state over the recorded data and identify the temporal occurrences of patterns similar to the ones experienced during the episodic events [28], [30].

## Supporting Information

Figure S1 The unpaired CS did not induce trace conditioning memory, whereas paired CS produced robust trace conditioning memory as assessed in the one-hour trace memory test. n = 10 mice for paired and unpaired groups. *** p<0.001.
(0.04 MB JPG)

Figure S2 Confirming the position of recording electrodes in the CA1 region of the mouse hippocampus. (A) Histological confirmation of electrode placement. The top panel demonstrates the position of the electrodes with orange bars in the atlas of the mouse brain. The two examples (the middle and bottom panels) show Nissl staining in the hippocampal CA1 region from one mouse. The arrows indicate the locations of electrode tips in the CA1 pyramidal layers. (B) Physiological confirmation of electrode placement by the occurrence of ripples. Local field potentials (the upper panel) and the filtered ripples (the lower panel) were shown from ten recording channels.
(1.24 MB JPG)

Figure S3 Stable recordings were confirmed as judged by the waveforms and inter-spike interval (ISI) histograms of recorded cells. Eight representative putative pyramidal cells are shown here. (A)–(H) The left columns are waveforms and the right columns are inter-spike interval histograms. The waveforms were plotted from a 70-sec recording before (top row), during (middle row), and after trace-conditioning trials. A 10-sec recording for each trial was plotted. The ISIs were analyzed by using the corresponding data and the bin size is 0.005 s. (A)–(D) are the data recorded from steretrodes, and (E)–(H) are the data recorded from tetrodes.
(2.84 MB JPG)

Figure S4 Stable recordings for putative interneurons in the hippocampus. Waveforms and inter-spike interval histogram of interneurons (eight representative units are represented here). (A)–(H) The left columns are waveforms and the right columns are inter-spike interval histograms. The waveforms were plotted from a 70-sec recording before (top row), during (middle), and after trace-conditionings (bottom row). A 10-sec recording for each trial was plotted. The ISIs were analyzed by using the corresponding data and the bin size is 0.005 s. (A)–(D) are the data recorded from steretrodes, and (E)–(H) are the data recorded from tetrodes.
(3.06 MB JPG)

Figure S5 A spike-raster plot of simultaneously recorded 208 CA1 units from mouse #1. (A) The sixty-sec spike-raster plot demonstrates neural activity when a tone was delivered and then followed by a foot shock in the third trial during the training. Only a set of the 208 simultaneously recorded CA1 cells were shown here. Two 500-ms black parallel windows demonstrate the MDA sliding-windows for computing dynamic trajectories in MDA subspaces. (B) The averaged histograms show neural activity during training (the left column) and during trace recall (the right column) for the corresponding colored units shown in (A) across all trials. The histograms at the first row show that this group of neurons had significant prolonged increased responses to the US presentation as well as increased responses to the CS during training. At trace recall, they also had significant increased responses to the recall tone. But the variations in spike firings of these neurons could not allow the confident assessment of the traced retrieval, although we noted a blip around 24 seconds after the recall tone was delivered. The data were pooled from all trials. The histograms at the second row show that these US-responsive neurons increased their firing rates at both onset and offset of the tone during recall. Interestingly, this group seemed to have a double peak in responding to the recall tone. The histograms at the third row show that these US-responsive neurons had a smaller response to the CS during training, and they also did not show significant firing increases when the recall tone was delivered. The fourth-row (dark blue) histograms show that this group of neurons showed a transient increase in their firing rates responding to the US during training, and they also seemed to have transient responses to the tone during recall. The fifth-row histograms show that a small set of responsive neurons decreased their firing rates both to the US during training and the tone during recall. The last-row histograms show that non-responsive neurons had no significant responses to both the US and the CS during training, and no responses to the tone during recall. X axis is time; Y axis is responsive ratio of frequency over baseline firing rates.
(3.77 MB JPG)

Figure S6 Representative units show neural responses to the US and/or the CS over trials. (A) Spike rasters show firing activity of a US-responsive unit. Red spike rasters show the firing activity of a representative unit in response to the US conditioning; two rasters in brown show the tone-triggered responses in the first two training trials; the five rasters in purple show the tone-triggered responses in the last five training trials; the seven rasters in green show the tone-triggered responses of this unit in the seven trials during trace recall trials; the seven rasters in blue show the naïve tone-triggered responses before paring. Time is represented in seconds on the X axis, and the trial number is listed on the Y axis. (B) The frequency responses of the unit shown in (A), obtained by smoothing the spike counts through a Gaussian kernel, indicate that this unit significantly increased its firing rate only in response to the US. (C) Spike rasters show firing activity of a US/CS responsive unit in response to various stimuli. The seven rasters in red show the US-triggered responses in this unit over trials; the two rasters in brown show the tone-triggered responses during the first two training trials; the five rasters in purple show the tone-triggered responses in the last five training trials; the seven rasters in green show the tone-triggered responses during traced recall; the seven rasters in blue show that the naïve tone did not trigger significant responses before training. Time is represented on the X axis, and the trial number is listed on the Y axis. (D) The frequency responses of the unit shown in (C), obtained by smoothing the spike count through a Gaussian kernel, indicate that this unit significantly increased its firing rate in response to the US, the tone during training, and the tone during recall. Please note that the responses to contextual recall are not listed due to the lack of objective time points for setting time zero. The need for averaging the firing responses of a single unit over trials illustrates the importance of large-scale pattern classification for revealing patterns, at any given time point, from the simultaneously recorded cell population. (E) Hierarchical clustering of the responses of 1454 recorded CA1 neurons to fear conditioning. The data were pooled from six mice. Only 9 neurons showed responsiveness to the tone before learning (CSb), the tone during CS/US pairing (CSd), the tone at trace recall (CSa), and foot shock (US); 94 neurons responded to US, CSd and CSa, which was ∼6.5% of all recorded neurons; 24 neurons responded to US and CSa; 277 neurons responded to US and CSd, which constituted 19.1% of all recorded neurons; 265 neurons responded to US but not to tones; interestingly, 32 neurons responded only to CSb, without any significant responses to tones after pairing, or to the US; 66 neurons responded only to CSa (4.5% of all recorded neurons); the other remaining 47.2% neurons had no significant responses to all four types of stimuli. The color bar on the left indicates the normalized response magnitude.
(0.66 MB JPG)

Figure S7 Visualization of various CA1 ensemble encoding patterns and the transient dynamics in MDA subspaces. (A) MDA analysis shows CA1 ensemble representations of various episodic events in mouse #1. Please note that the naïve tone (prior to conditioning) overlapped significantly with the Rest cluster, indicating that the naïve tone did not trigger significant CA1 responses. (B) A rotated view of the MDA subspace shown in (A) demonstrates that the ensemble representations of these events were well separated. In MDA spaces, the discrete dots in various shapes represent firing patterns in response to a give type of stimulus (e.g. CS, or US, etc); color ellipsoids were constructed for representing neural ensemble responses to various kinds of stimuli by fitting Gaussian distribution to the projected points (discrete dots) for each class. (C) Two representative US-CS association trajectory traces at the third (blue) and fifth learning trials (magenta), respectively, are shown here. At these learning stages, the foot shock triggered the patterns that moved from the Rest state to the US cluster, and then directly visited and hovered around the CS for a while before returning to the Rest. (D) A rotated view of MDA subspace in (A) shows the reliable moving paths of two activation traces despite the variability at the single neuron level.
(0.45 MB JPG)

Figure S8 Relationship between ripples and pattern retrievals during the freezing state of contextual recall. (A) A representative spectrogram during freezing is shown in the left panel. One typical ripple oscillation is shown on a finer time scale in the top right panel. It was filtered out from the recorded field potential using a 100–250 Hz band-pass filter (the bottom right panel). (B) The power spectral density plot shows a significant peak in 150–250 Hz range during freezing (red). It was absent when the animal was in the active exploration state (blue). (C) Three retrieved CS ensemble traces were plotted here (in different colors) during the freezing state of contextual recall. (D) Three retrieved US ensemble traces were plotted during the freezing state of contextual recall. (E) An example of co-occurrence of hippocampal ripples and a CS ensemble trajectory is shown. The red line about the upper ripple oscillations indicates the threshold line (5 s.d. above mean power). The blue curve here corresponds to the blue trajectory shown in (C). The projection distance of the CS trajectory was measured along the line through the Rest and CS ellipsoid centers. (F) Co-occurrence of ripples and a US pattern is illustrated here. The dynamic trajectory is the same as the blue trajectory in (D). The projection distance of the CS trajectory was measured along the line through the Rest and US ellipsoid centers. (G) Occurrence distribution of retrieved three major types of ensemble patterns in relation with ripples (±1-sec time window from the peak center of each ripple) during the contextual recall freezing state. (H) Occurrence distribution of large-amplitude ripples (5 s.d. above mean power) in relation with retrieved ensemble patterns (±1-sec time window from the peak of each trajectory). About 49.2% of ensemble pattern retrievals were accompanied with large-amplitude ripples.
(0.53 MB JPG)

Figure S9 (A) Seven colored curves show the stable ensemble trajectories during the seven trace recall trials in mouse #1. The tone-triggered the peak responses within 200 mini-seconds. The distance is computed by projecting activity trajectories onto the line through both the centers of the CS ellipsoid and Rest ellipsoid. (B) Seven circles show the time latency to freezing after offset of the recall-tone traces during each of the seven recall trials. Color corresponds to the trial number in (A). On average, the latency is 1.9857±1.5137 sec for this animal.
(0.17 MB JPG)

Figure S10 Diverse reactivation patterns in CA1 during trace memory recall. (A–D) Four distinct trajectories are separately shown in MDA subspaces. These patterns occurred during the 1-min trace recall period. (E–H) Rotated views of the same set of the trajectories in MDA subspaces shown in (A–D). The same trace is listed in the same row, side by side.
(0.42 MB JPG)

Table S1 Robustness of the MDA statistical classification.
(0.03 MB DOC)<>
