# Supplementary figures and images for: Neural Population-Level Memory Traces in the Mouse Hippocampus
Source: PLoS One. 2009 Dec 16;4(12):e8256. doi: 10.1371/journal.pone.0008256 (PMC2788416; doi:10.1371/journal.pone.0008256)

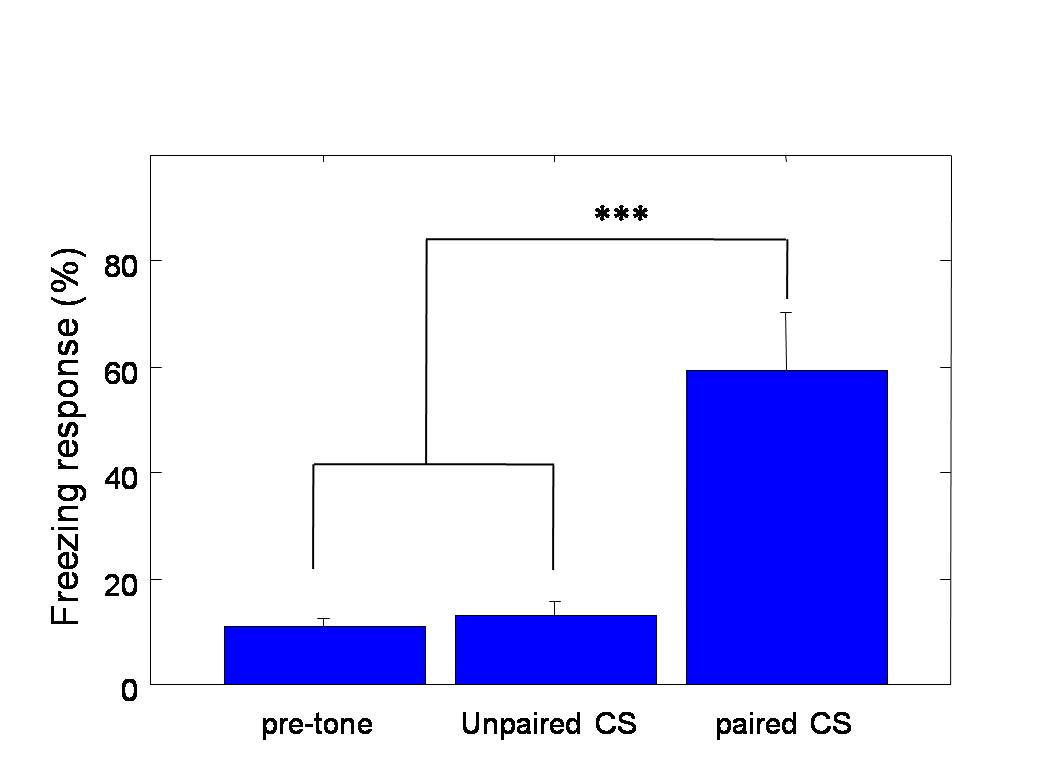

Supplement: Figure S1 — The unpaired CS did not induce trace conditioning memory, whereas paired CS produced robust trace conditioning memory as assessed in the one-hour trace memory test. n = 10 mice for paired and unpaired groups. *** p<0.001. (0.04 MB JPG) [file pone.0008256.s001.jpg]

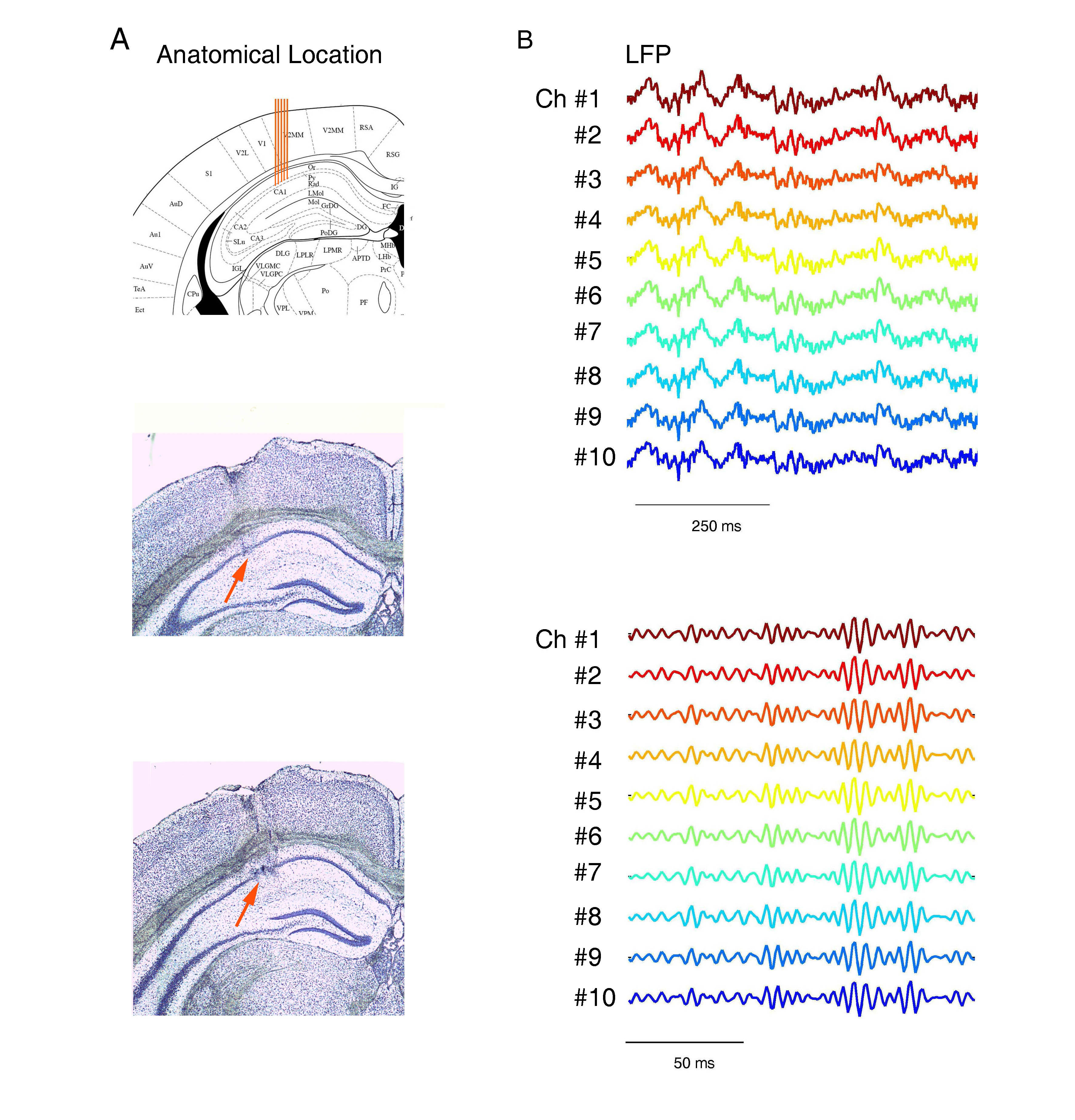

Supplement: Figure S2 — Confirming the position of recording electrodes in the CA1 region of the mouse hippocampus. (A) Histological confirmation of electrode placement. The top panel demonstrates the position of the electrodes with orange bars in the atlas of the mouse brain. The two examples (the middle and bottom panels) show Nissl staining in the hippocampal CA1 region from one mouse. The arrows indicate the locations of electrode tips in the CA1 pyramidal layers. (B) Physiological confirmation of electrode placement by the occurrence of ripples. Local field potentials (the upper panel) and the filtered ripples (the lower panel) were shown from ten recording channels. (1.24 MB JPG) [file pone.0008256.s002.jpg]

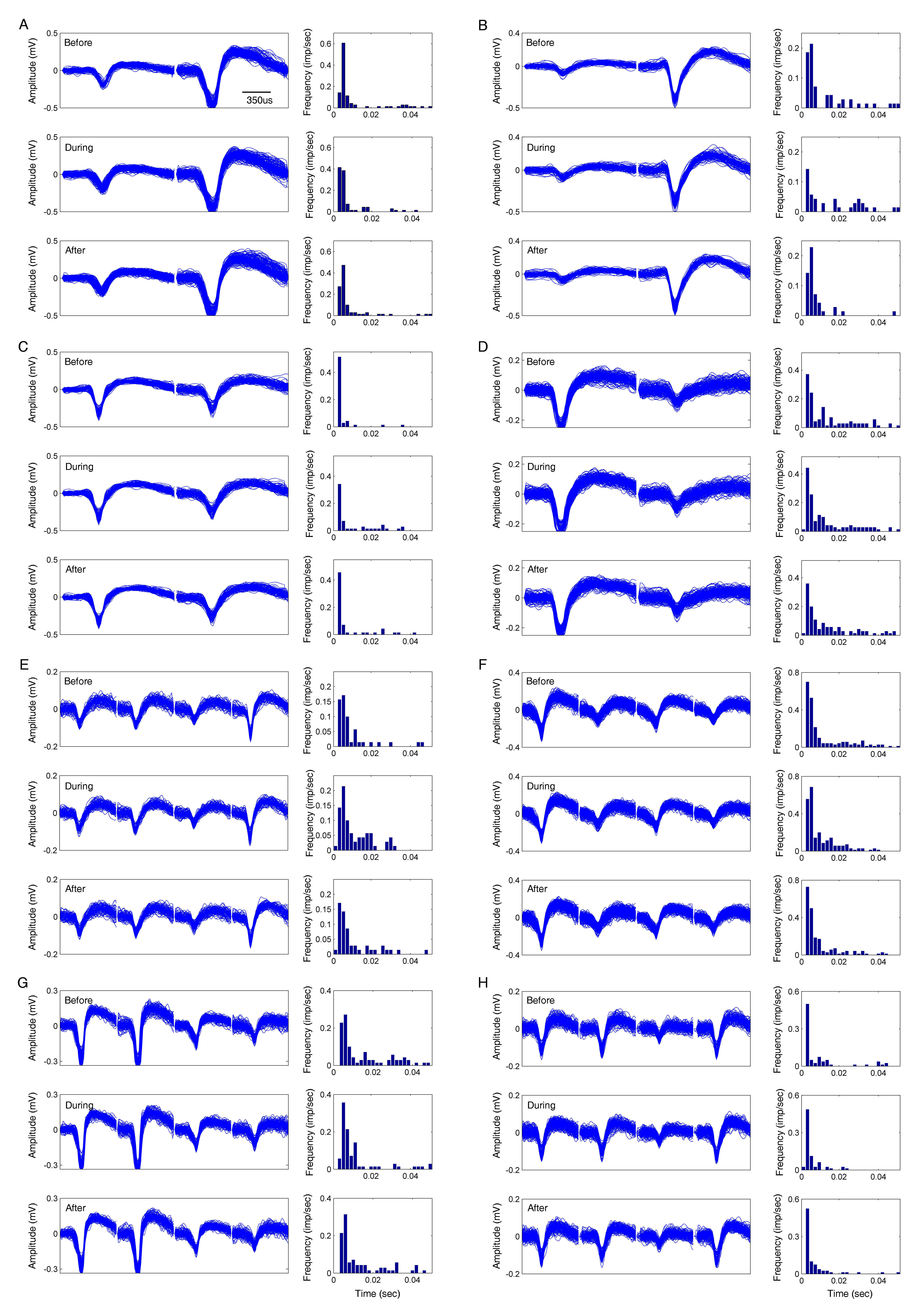

Supplement: Figure S3 — Stable recordings were confirmed as judged by the waveforms and inter-spike interval (ISI) histograms of recorded cells. Eight representative putative pyramidal cells are shown here. (A)–(H) The left columns are waveforms and the right columns are inter-spike interval histograms. The waveforms were plotted from a 70-sec recording before (top row), during (middle row), and after trace-conditioning trials. A 10-sec recording for each trial was plotted. The ISIs were analyzed by using the corresponding data and the bin size is 0.005 s. (A)–(D) are the data recorded from steretrodes, and (E)–(H) are the data recorded from tetrodes. (2.84 MB JPG) [file pone.0008256.s003.jpg]

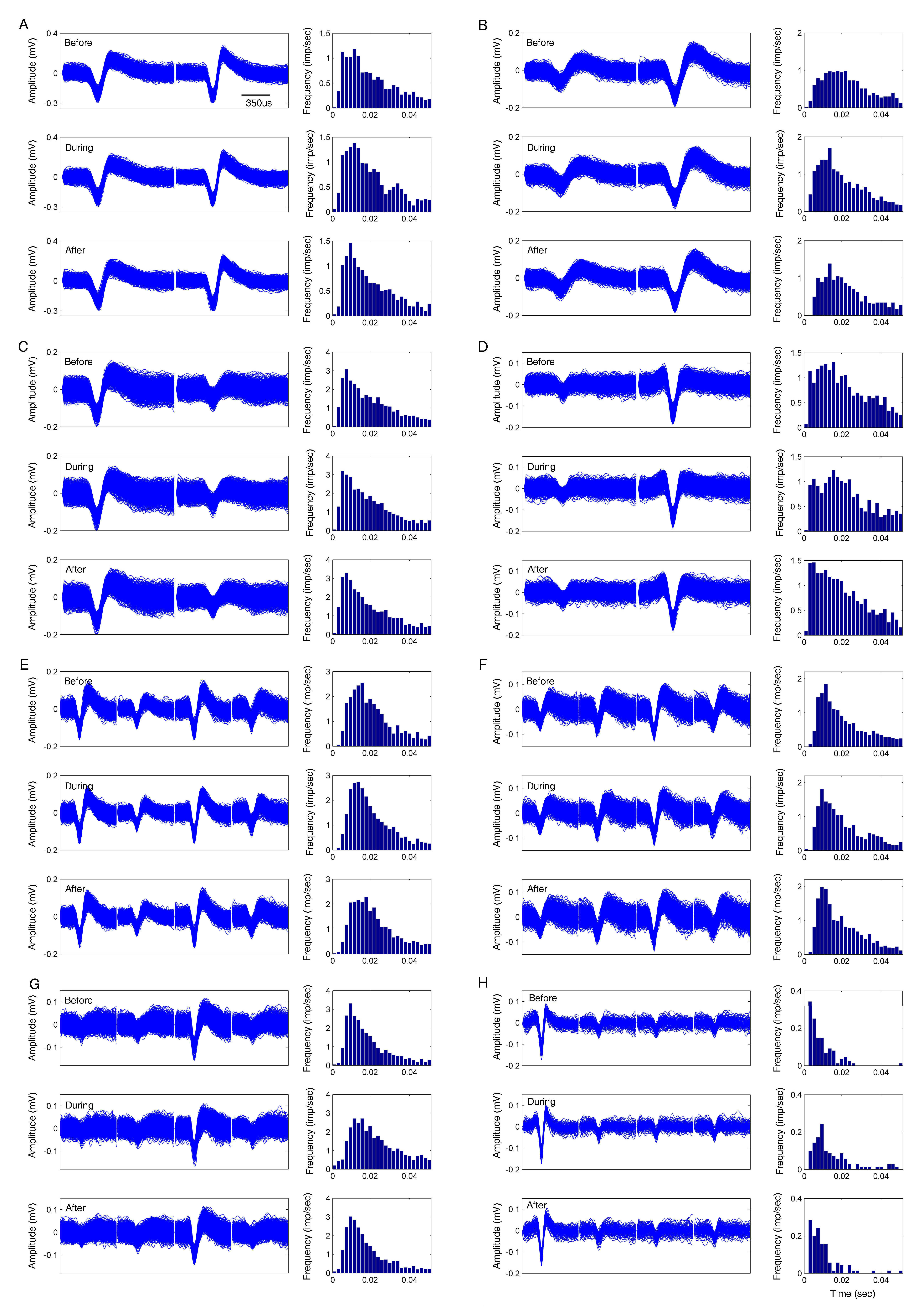

Supplement: Figure S4 — Stable recordings for putative interneurons in the hippocampus. Waveforms and inter-spike interval histogram of interneurons (eight representative units are represented here). (A)–(H) The left columns are waveforms and the right columns are inter-spike interval histograms. The waveforms were plotted from a 70-sec recording before (top row), during (middle), and after trace-conditionings (bottom row). A 10-sec recording for each trial was plotted. The ISIs were analyzed by using the corresponding data and the bin size is 0.005 s. (A)–(D) are the data recorded from steretrodes, and (E)–(H) are the data recorded from tetrodes. (3.06 MB JPG) [file pone.0008256.s004.jpg]

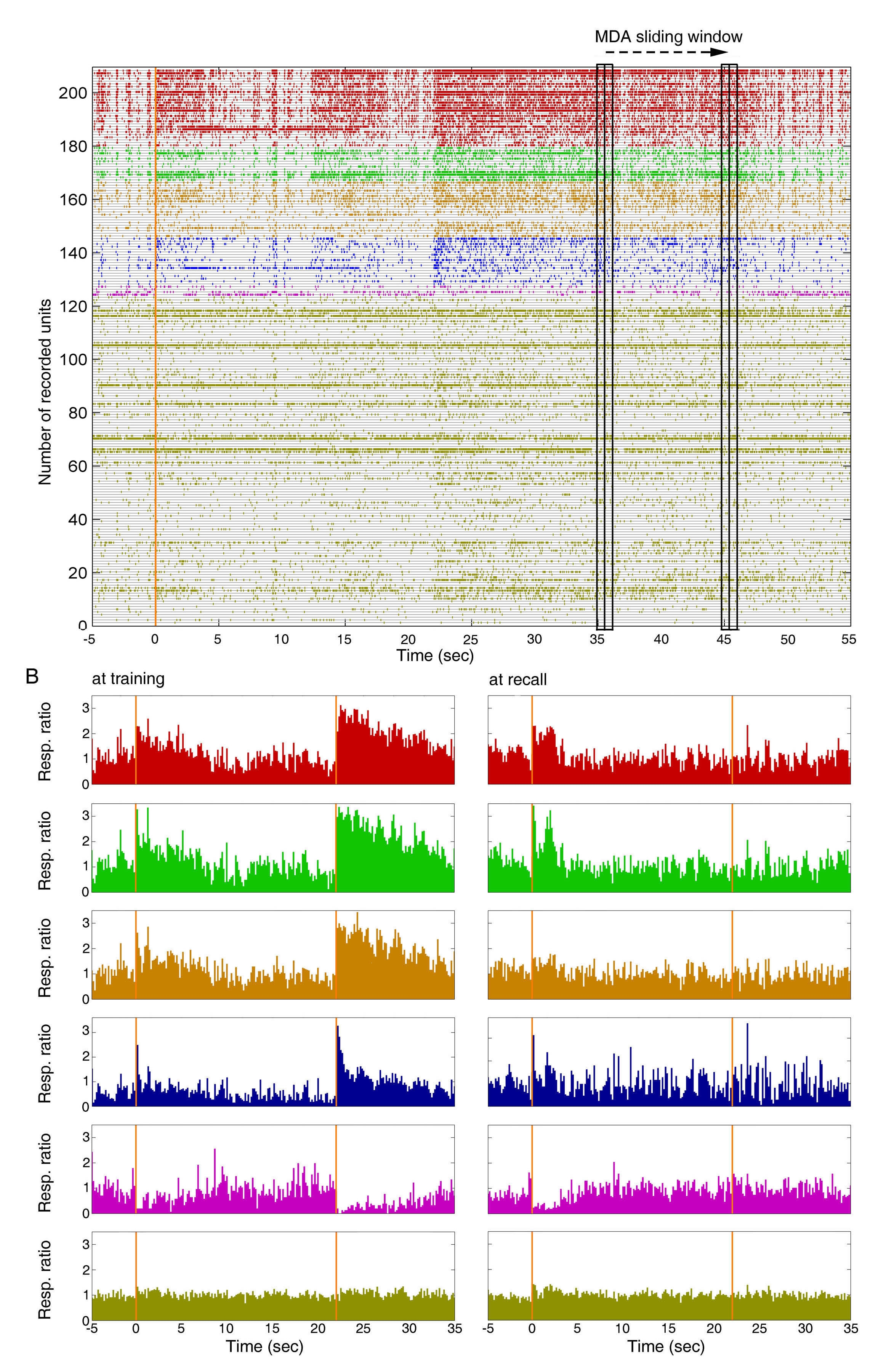

Supplement: Figure S5 — A spike-raster plot of simultaneously recorded 208 CA1 units from mouse #1. (A) The sixty-sec spike-raster plot demonstrates neural activity when a tone was delivered and then followed by a foot shock in the third trial during the training. Only a set of the 208 simultaneously recorded CA1 cells were shown here. Two 500-ms black parallel windows demonstrate the MDA sliding-windows for computing dynamic trajectories in MDA subspaces. (B) The averaged histograms show neural activity during training (the left column) and during trace recall (the right column) for the corresponding colored units shown in (A) across all trials. The histograms at the first row show that this group of neurons had significant prolonged increased responses to the US presentation as well as increased responses to the CS during training. At trace recall, they also had significant increased responses to the recall tone. But the variations in spike firings of these neurons could not allow the confident assessment of the traced retrieval, although we noted a blip around 24 seconds after the recall tone was delivered. The data were pooled from all trials. The histograms at the second row show that these US-responsive neurons increased their firing rates at both onset and offset of the tone during recall. Interestingly, this group seemed to have a double peak in responding to the recall tone. The histograms at the third row show that these US-responsive neurons had a smaller response to the CS during training, and they also did not show significant firing increases when the recall tone was delivered. The fourth-row (dark blue) histograms show that this group of neurons showed a transient increase in their firing rates responding to the US during training, and they also seemed to have transient responses to the tone during recall. The fifth-row histograms show that a small set of responsive neurons decreased their firing rates both to the US during training and the tone during recall. The last-row h [file pone.0008256.s005.jpg]

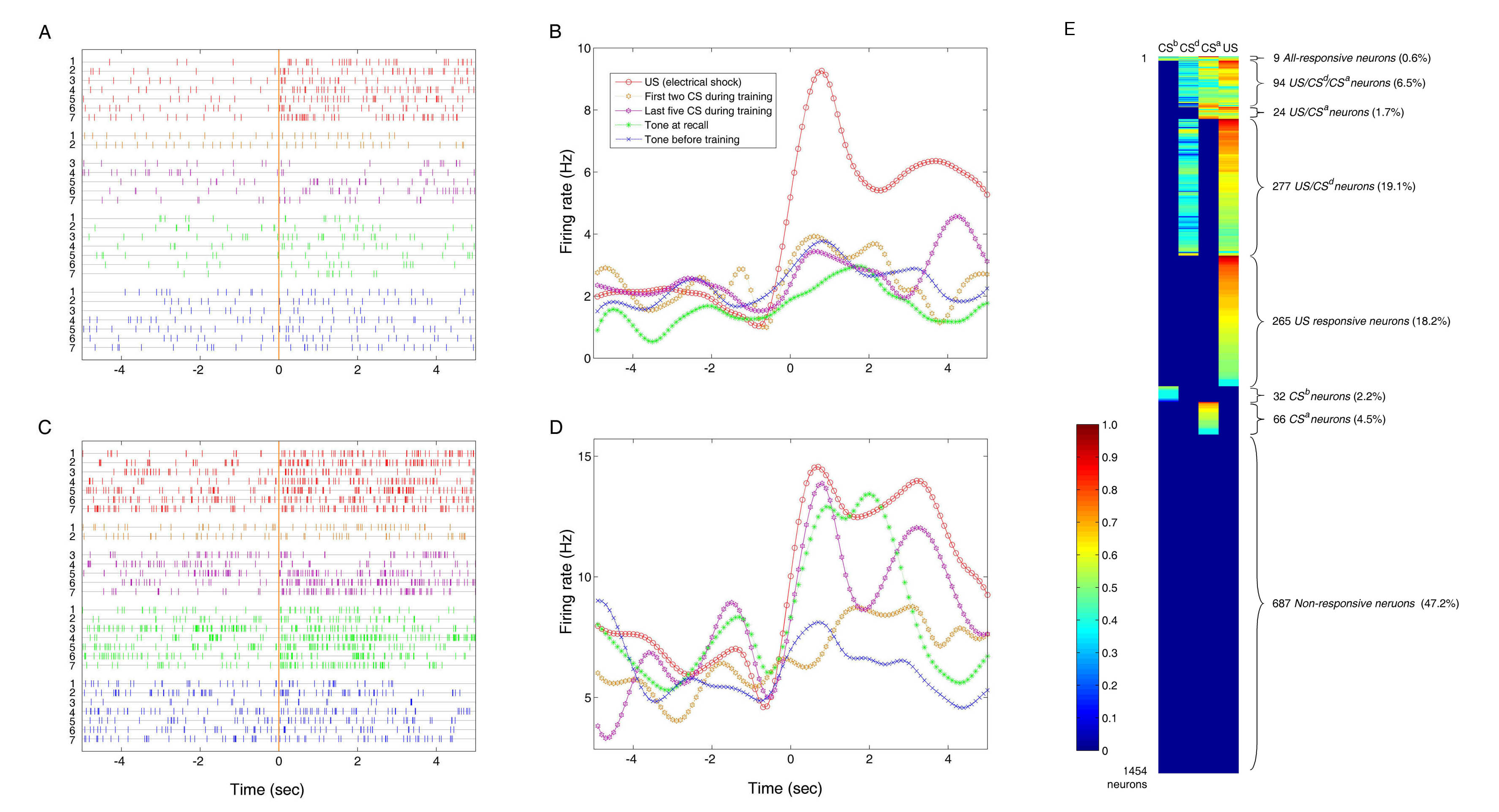

Supplement: Figure S6 — Representative units show neural responses to the US and/or the CS over trials. (A) Spike rasters show firing activity of a US-responsive unit. Red spike rasters show the firing activity of a representative unit in response to the US conditioning; two rasters in brown show the tone-triggered responses in the first two training trials; the five rasters in purple show the tone-triggered responses in the last five training trials; the seven rasters in green show the tone-triggered responses of this unit in the seven trials during trace recall trials; the seven rasters in blue show the naïve tone-triggered responses before paring. Time is represented in seconds on the X axis, and the trial number is listed on the Y axis. (B) The frequency responses of the unit shown in (A), obtained by smoothing the spike counts through a Gaussian kernel, indicate that this unit significantly increased its firing rate only in response to the US. (C) Spike rasters show firing activity of a US/CS responsive unit in response to various stimuli. The seven rasters in red show the US-triggered responses in this unit over trials; the two rasters in brown show the tone-triggered responses during the first two training trials; the five rasters in purple show the tone-triggered responses in the last five training trials; the seven rasters in green show the tone-triggered responses during traced recall; the seven rasters in blue show that the naïve tone did not trigger significant responses before training. Time is represented on the X axis, and the trial number is listed on the Y axis. (D) The frequency responses of the unit shown in (C), obtained by smoothing the spike count through a Gaussian kernel, indicate that this unit significantly increased its firing rate in response to the US, the tone during training, and the tone during recall. Please note that the responses to contextual recall are not listed due to the lack of objective time points for setting time zero. The need for averaging the [file pone.0008256.s006.jpg]

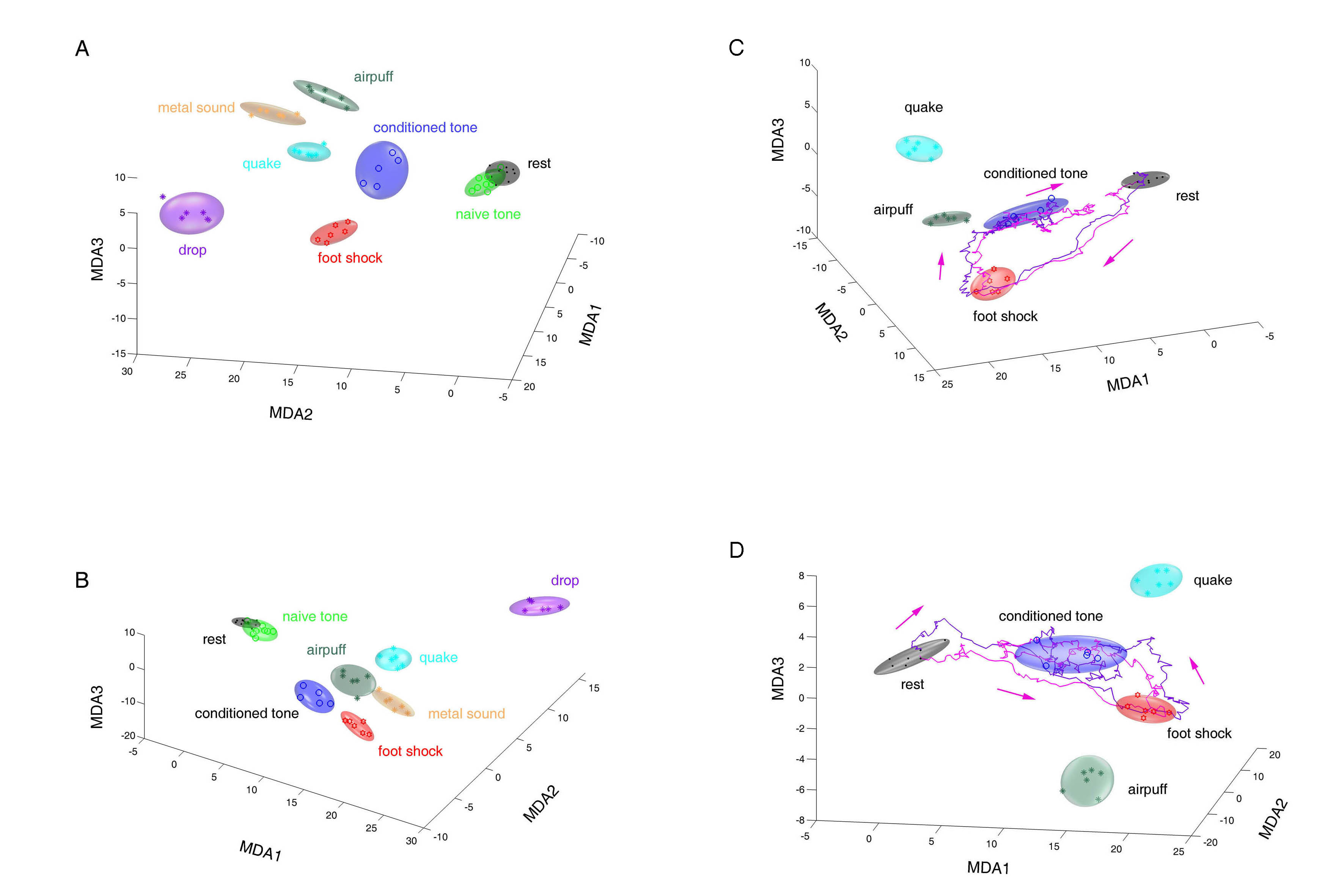

Supplement: Figure S7 — Visualization of various CA1 ensemble encoding patterns and the transient dynamics in MDA subspaces. (A) MDA analysis shows CA1 ensemble representations of various episodic events in mouse #1. Please note that the naïve tone (prior to conditioning) overlapped significantly with the Rest cluster, indicating that the naïve tone did not trigger significant CA1 responses. (B) A rotated view of the MDA subspace shown in (A) demonstrates that the ensemble representations of these events were well separated. In MDA spaces, the discrete dots in various shapes represent firing patterns in response to a give type of stimulus (e.g. CS, or US, etc); color ellipsoids were constructed for representing neural ensemble responses to various kinds of stimuli by fitting Gaussian distribution to the projected points (discrete dots) for each class. (C) Two representative US-CS association trajectory traces at the third (blue) and fifth learning trials (magenta), respectively, are shown here. At these learning stages, the foot shock triggered the patterns that moved from the Rest state to the US cluster, and then directly visited and hovered around the CS for a while before returning to the Rest. (D) A rotated view of MDA subspace in (A) shows the reliable moving paths of two activation traces despite the variability at the single neuron level. (0.45 MB JPG) [file pone.0008256.s007.jpg]

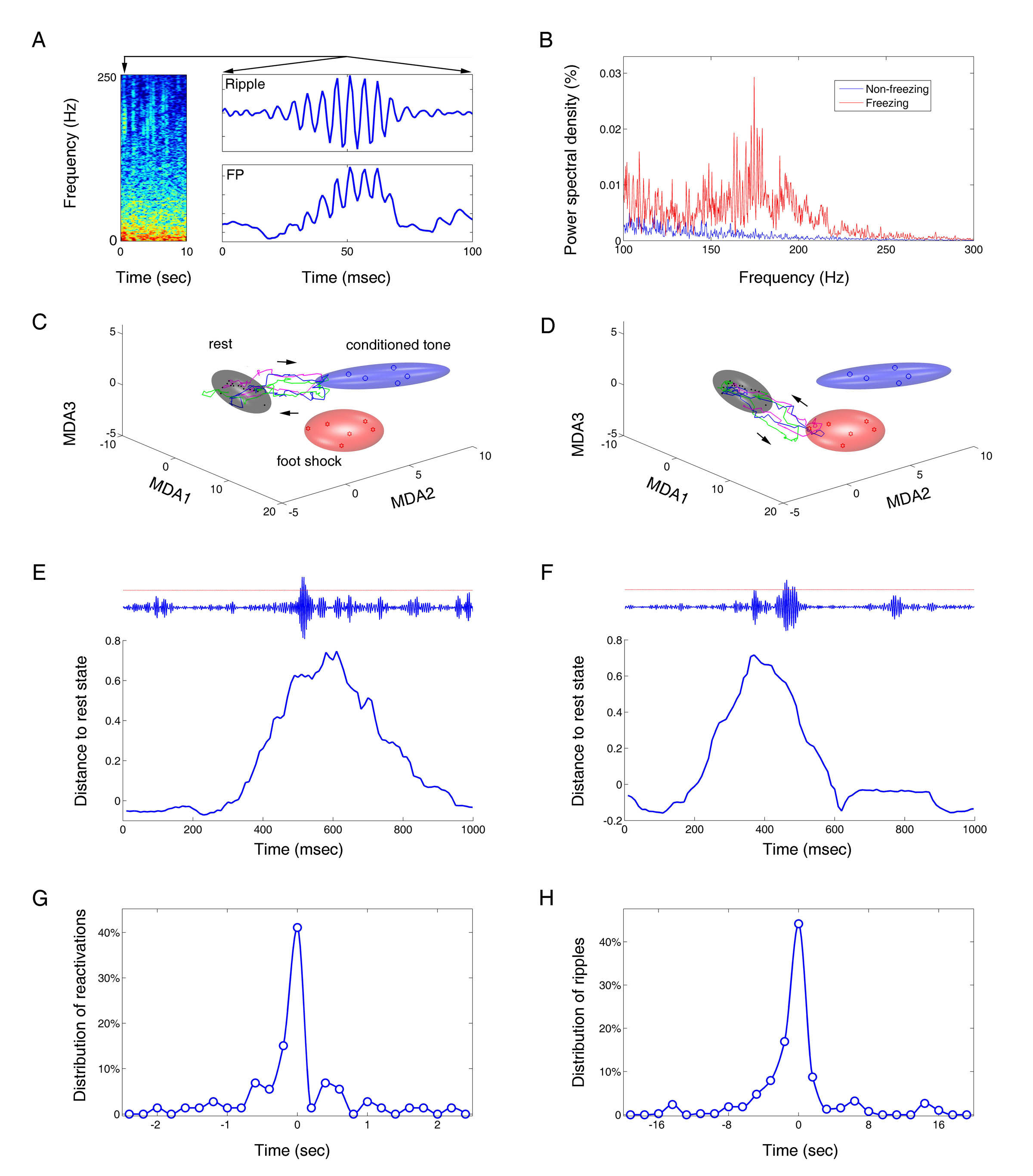

Supplement: Figure S8 — Relationship between ripples and pattern retrievals during the freezing state of contextual recall. (A) A representative spectrogram during freezing is shown in the left panel. One typical ripple oscillation is shown on a finer time scale in the top right panel. It was filtered out from the recorded field potential using a 100–250 Hz band-pass filter (the bottom right panel). (B) The power spectral density plot shows a significant peak in 150–250 Hz range during freezing (red). It was absent when the animal was in the active exploration state (blue). (C) Three retrieved CS ensemble traces were plotted here (in different colors) during the freezing state of contextual recall. (D) Three retrieved US ensemble traces were plotted during the freezing state of contextual recall. (E) An example of co-occurrence of hippocampal ripples and a CS ensemble trajectory is shown. The red line about the upper ripple oscillations indicates the threshold line (5 s.d. above mean power). The blue curve here corresponds to the blue trajectory shown in (C). The projection distance of the CS trajectory was measured along the line through the Rest and CS ellipsoid centers. (F) Co-occurrence of ripples and a US pattern is illustrated here. The dynamic trajectory is the same as the blue trajectory in (D). The projection distance of the CS trajectory was measured along the line through the Rest and US ellipsoid centers. (G) Occurrence distribution of retrieved three major types of ensemble patterns in relation with ripples (±1-sec time window from the peak center of each ripple) during the contextual recall freezing state. (H) Occurrence distribution of large-amplitude ripples (5 s.d. above mean power) in relation with retrieved ensemble patterns (±1-sec time window from the peak of each trajectory). About 49.2% of ensemble pattern retrievals were accompanied with large-amplitude ripples. (0.53 MB JPG) [file pone.0008256.s008.jpg]

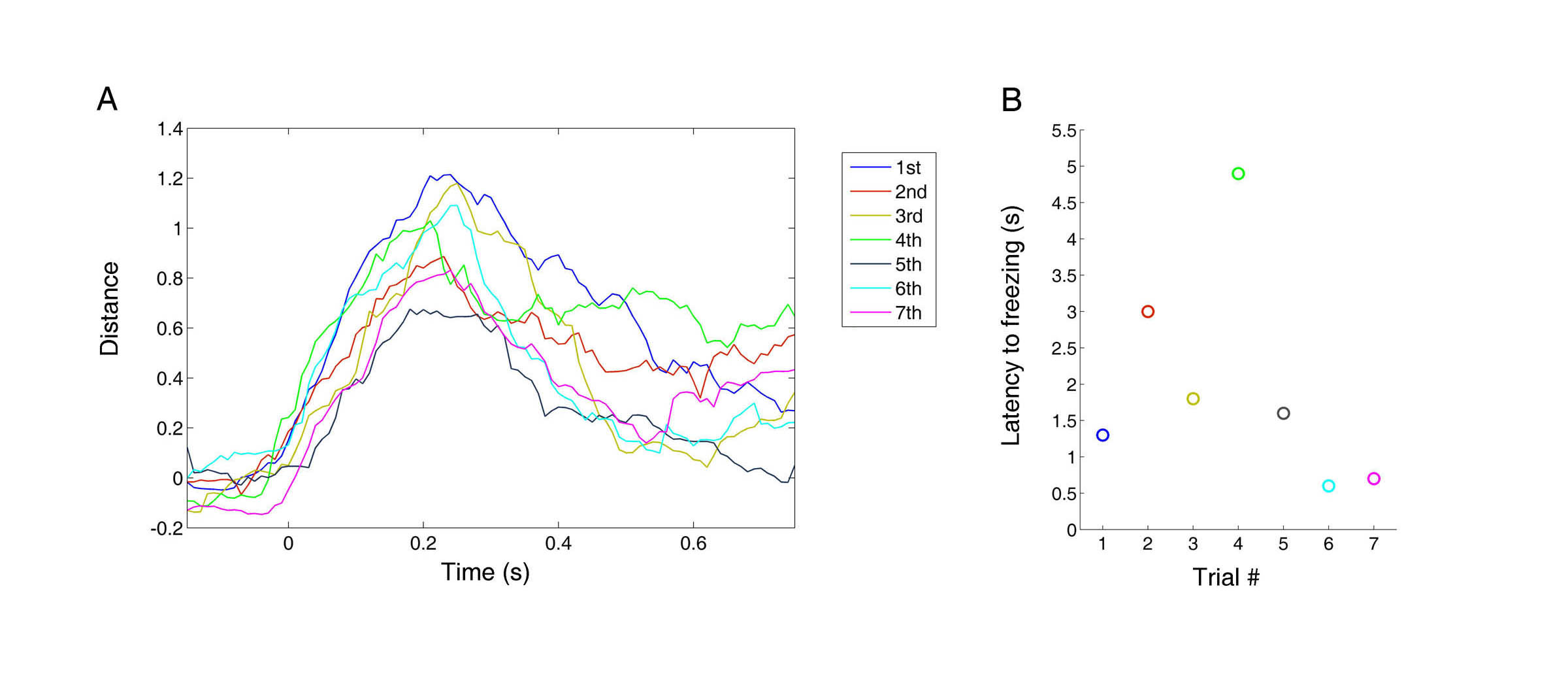

Supplement: Figure S9 — (A) Seven colored curves show the stable ensemble trajectories during the seven trace recall trials in mouse #1. The tone-triggered the peak responses within 200 mini-seconds. The distance is computed by projecting activity trajectories onto the line through both the centers of the CS ellipsoid and Rest ellipsoid. (B) Seven circles show the time latency to freezing after offset of the recall-tone traces during each of the seven recall trials. Color corresponds to the trial number in (A). On average, the latency is 1.9857±1.5137 sec for this animal. (0.17 MB JPG) [file pone.0008256.s009.jpg]

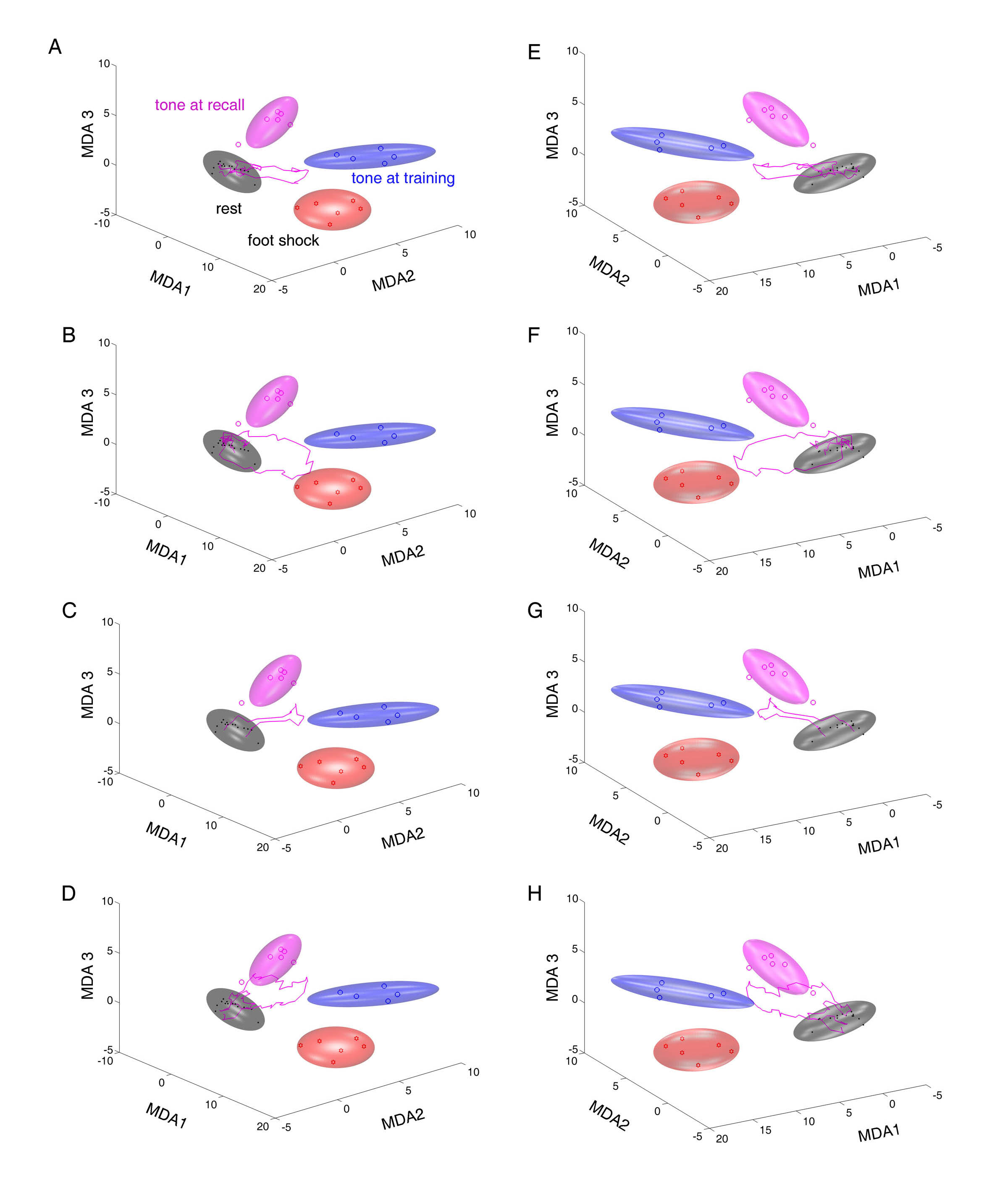

Supplement: Figure S10 — Diverse reactivation patterns in CA1 during trace memory recall. (A–D) Four distinct trajectories are separately shown in MDA subspaces. These patterns occurred during the 1-min trace recall period. (E–H) Rotated views of the same set of the trajectories in MDA subspaces shown in (A–D). The same trace is listed in the same row, side by side. (0.42 MB JPG) [file pone.0008256.s010.jpg]
