# Supplementary material for: Neural Population-Level Memory Traces in the Mouse Hippocampus
Source: PLoS One. 2009 Dec 16;4(12):e8256. doi: 10.1371/journal.pone.0008256 (PMC2788416; doi:10.1371/journal.pone.0008256)
Supplement: Table S1 — Robustness of the MDA statistical classification. (0.03 MB DOC)<> [file pone.0008256.s011.doc]

**Table S1. Robustness of the MDA statistical classification.** Different partitions of the data as training versus testing data allowed the assessment of validity of MDA classification. All of the six datasets from six recorded mice using “leave-one-out” method are presented below. CSb (Tone before conditioning); CSd (Tone during conditioning); CSa (Tone at recall).

| Mouse# | Rest | CSb | CSd | CSa | US |
| --- | --- | --- | --- | --- | --- |
| 1 | 84 | 99 | 99 | 99 | 99 |
| 2 | 92 | 98 | 85 | 99 | 99 |
| 3 | 84 | 89 | 85 | 99 | 99 |
| 4 | 91 | 99 | 99 | 99 | 99 |
| 5 | 88 | 98 | 99 | 99 | 99 |
| 6 | 86 | 99 | 85 | 99 | 99 |
